# Predicting global distributions of eukaryotic plankton communities from satellite data

**DOI:** 10.1038/s43705-023-00308-7

**Published:** 2023-09-22

**Authors:** Hiroto Kaneko, Hisashi Endo, Nicolas Henry, Cédric Berney, Frédéric Mahé, Julie Poulain, Karine Labadie, Odette Beluche, Roy El Hourany, Tara Oceans Coordinators, Silvia G. Acinas, Marcel Babin, Peer Bork, Chris Bowler, Guy Cochrane, Colomban de Vargas, Gabriel Gorsky, Lionel Guidi, Nigel Grimsley, Pascal Hingamp, Daniele Iudicone, Olivier Jaillon, Stefanie Kandels, Eric Karsenti, Fabrice Not, Nicole Poulton, Stéphane Pesant, Christian Sardet, Sabrina Speich, Lars Stemmann, Matthew B. Sullivan, Shinichi Sunagawa, Samuel Chaffron, Patrick Wincker, Ryosuke Nakamura, Lee Karp-Boss, Emmanuel Boss, Chris Bowler, Colomban de Vargas, Kentaro Tomii, Hiroyuki Ogata

**Affiliations:** 1 Institute for Chemical Research, Kyoto University, Uji, Kyoto Japan; 2 CNRS, Sorbonne Université, FR2424, ABiMS, Station Biologique de Roscoff, 29680 Roscoff, France; 3 Research Federation for the study of Global Ocean Systems Ecology and Evolution, FR2022/Tara GOSEE, 75016 Paris, France; 4 Sorbonne Université, CNRS, Station Biologique de Roscoff, UMR7144, ECOMAP, 29680 Roscoff, France; 5 CIRAD, UMR PHIM, F-34398 Montpellier, France; 6 PHIM, Univ Montpellier, CIRAD, INRAE, Institut Agro, IRD, Montpellier, France; 7 Génomique Métabolique, Genoscope, Institut François Jacob, CEA, CNRS, Univ Evry, Université Paris-Saclay, 2 Rue Gaston Crémieux, 91057 Evry, France; 8 Genoscope, Institut François Jacob, Commissariat à l’Energie Atomique (CEA), Université Paris-Saclay, 2 Rue Gaston Crémieux, 91057 Evry, France; 9 Univ. Littoral Côte d’Opale, Univ. Lille, CNRS, IRD, UMR 8187, LOG, Laboratoire d’Océanologie et de Géosciences, F 62930 Wimereux, France; 10 Institut de Biologie de l’Ecole Normale Supérieure (IBENS), Ecole Normale Supérieure, CNRS, INSERM, Université PSL, 75005 Paris, France; 11 Nantes Université, École Centrale Nantes, CNRS, LS2N, UMR 6004, F-44000 Nantes, France; 12 Digital Architecture Research Center, National Institute of Advanced Industrial Science and Technology (AIST), Tokyo, Japan; 13 School of Marine Sciences, University of Maine, Orono, 04469 ME USA; 14 Artificial Intelligence Research Center, National Institute of Advanced Industrial Science and Technology (AIST), Tokyo, Japan; 15 Department of Marine Biology and Oceanography, Institut de Ciències del Mar (CSIC), Barcelona, Catalonia Spain; 16 Département de biologie, Québec Océan and Takuvik Joint International Laboratory (UMI3376), Université Laval (Canada) - CNRS (France), Université Laval, Québec, QC G1V 0A6 Canada; 17 Structural and Computational Biology, European Molecular Biology Laboratory, Meyerhofstrasse 1, 69117 Heidelberg, Germany; 18 Max Delbrück Centre for Molecular Medicine, 13125 Berlin, Germany; 19 Department of Bioinformatics, Biocenter, University of Würzburg, 97074 Würzburg, Germany; 20 European Molecular Biology Laboratory, European Bioinformatics Institute (EMBL-EBI), Welcome Trust Genome Campus, Hinxton, Cambridge UK; 21 CNRS, UMR 7144, EPEP & Sorbonne Universités, UPMC Université Paris 06, Station Biologique de Roscoff, 29680 Roscoff, France; 22 Sorbonne Université, UMR7093 Laboratoire d’océanographie de Villefranche (LOV), Institut de la Mer de Villefranche (IMEV), 06230 Villefranche-sur-Mer, France; 23 Department of Oceanography, University of Hawaii, Honolulu, HI 96822 USA; 24 CNRS, UMR 7232, BIOM, Avenue de Pierre Fabre, 66650 Banyuls-sur-Mer, France; 25 Sorbonne Universités Paris 06, OOB UPMC, Avenue de Pierre Fabre, 66650 Banyuls-sur-Mer, France; 26 Aix Marseille Univ, Université de Toulon, CNRS, IRD, MIO, Marseille, France; 27 Stazione Zoologica Anton Dohrn, Villa Comunale, 80121 Naples, Italy; 28 European Molecular Biology Laboratory Meyerhofstr. 1, 69117 Heidelberg, Germany; 29 Bigelow Laboratory for Ocean Sciences, East Boothbay, ME 04544 USA; 30 European Molecular Biology Laboratory, European Bioinformatics Institute, Wellcome Genome Campus, Hinxton, Cambridge CB10 1SD UK; 31 CNRS, UMR 7009 Biodev, Observatoire Océanologique, F-06230 Villefranche-sur-mer, France; 32 Laboratoire de Physique des Océans, UBO-IUEM, Place Copernic, 29820 Plouzané, France; 33 Department of Geosciences, Laboratoire de Météorologie Dynamique (LMD), Ecole Normale Supérieure, 24 rue Lhomond, 75231 Paris Cedex 05, France; 34 Department of Microbiology, The Ohio State University, Columbus, OH 43214 USA; 35 Department of Civil, Environmental and Geodetic Engineering, The Ohio State University, Columbus, OH 43214 USA; 36 Department of Biology, Institute of Microbiology and Swiss Institute of Bioinformatics, ETH Zurich, Vladimir-Prelog-Weg 4, 8093 Zurich, Switzerland

**Keywords:** Biooceanography, Microbial ecology

## Abstract

Satellite remote sensing is a powerful tool to monitor the global dynamics of marine plankton. Previous research has focused on developing models to predict the size or taxonomic groups of phytoplankton. Here, we present an approach to identify community types from a global plankton network that includes phytoplankton and heterotrophic protists and to predict their biogeography using global satellite observations. Six plankton community types were identified from a co-occurrence network inferred using a novel rDNA 18 S V4 planetary-scale eukaryotic metabarcoding dataset. Machine learning techniques were then applied to construct a model that predicted these community types from satellite data. The model showed an overall 67% accuracy in the prediction of the community types. The prediction using 17 satellite-derived parameters showed better performance than that using only temperature and/or the concentration of chlorophyll *a*. The constructed model predicted the global spatiotemporal distribution of community types over 19 years. The predicted distributions exhibited strong seasonal changes in community types in the subarctic–subtropical boundary regions, which were consistent with previous field observations. The model also identified the long-term trends in the distribution of community types, which suggested responses to ocean warming.

## Introduction

Monitoring the global dynamics of marine plankton is essential to understand the function of the marine microbial ecosystem and its interaction and evolution with climate change. Monitoring can also facilitate the discovery of new plankton species. Global plankton samples at a high spatial and temporal density using research ships alone cannot be obtained, owing to the extent of the ocean. However, regular and global remote sensing using satellites can potentially be used to solve this problem. The spectrum of light reflected from the ocean surface that is observed by satellites (remote sensing reflectance) has a specific relationship with plankton composition because some plankton species harbor pigments that absorb light. Environmental parameters, such as sea surface temperature (SST), are also related to plankton composition [1].

Several models for predicting plankton communities using satellite-derived data have been developed over the past decades [2, 3]. Most have focused on phytoplankton because these species always contain pigments, such as chlorophylls, carotenoids, and phycobilins, to capture light energy for photosynthesis [4]. The abundances of three size classes—micro-phytoplankton (>20 µm), nano-phytoplankton (2–20 µm) and pico-phytoplankton (0.2–2 µm)—can be predicted with simple models integrating only the concentration of chlorophyll *a* (Chl *a*), which is the core of the photosynthetic unit [5–7]. More advanced models have also been developed to predict size classes using remote sensing reflectance [8–11]. The abundance of taxonomic groups of phytoplankton is another target for predictive models. The abundance of diatoms, prymnesiophytes (haptophytes), green algae, and *Prochlorococcus* can be predicted using Chl *a* [5]. The PhytoDOAS model uses remote sensing reflectance data at high spectral resolution to predict the abundance of coccolithophores, dinoflagellates, cyanobacteria, and diatoms [12, 13]. Models also have been developed to predict the plankton communities. The PHYSAT model can predict communities dominated by diatoms, haptophytes, *Prochlorococcus*, and *Synechococcus* defined by the pigment concentration ratio [14, 15]. Another model has been developed to predict the distribution of biogeochemical provinces [16].

Despite these advantages, these previous methods have a limitation with regard to the number of defined plankton groups because most are based on empirical relationships between pigments and light absorption. Although these methods provide a synoptic view of the spatiotemporal extent of the main groups of phytoplankton, they lack taxonomic resolution and cannot reproduce the complexity of a planktonic community. To tackle this point, this study presents a machine-learning model for the satellite-based prediction of the global distribution of the community types captured by an ecological network of plankton. Its target was a community composed of phytoplankton and heterotrophic protists delineated from rDNA 18 S V4 metabarcoding data at a high taxonomic resolution. We used a network-oriented approach, which was inspired by the Bayesian network model used to predict metabarcoding-based bacterial composition in the English Channel [17]. There are two difficulties in predicting species composition directly from satellite-derived data. The first difficulty is the substantial number of response variables compared with predictor variables. There are hundreds of species represented in the metabarcoding dataset (after selection by their occurrence) but only 17 parameters of ocean color data acquired by multispectral sensors are available as predictor variables. The second difficulty is the small number of samples. In this study, we used the largest available compilation of eukaryotic metabarcoding data, complemented with novel sequence data from the *Tara* Oceans expeditions, but only a few hundred samples were available for analysis after appropriate filtering. Focusing on ecological networks alleviated these two difficulties by reducing the number of variables (dimensionality) in the metabarcoding data. Ecological networks tend to be structured and are non-randomly assembled [18]. Indeed, a previous study showed that, through an unsupervised approach for community delineation, the global plankton network is self-organized by marine biomes [19]. We took advantage of this property of plankton networks to reduce dimensionality and convert the problem into a multiclass prediction.

## Materials and methods

### Satellite data

Ocean color data acquired by the Moderate Resolution Imaging Spectroradiometer on board the Aqua and the Terra satellites were used in this study. Level-3 data, mapped to a 5ʹ (ca. 9 km on the Equator) square monthly grid, were downloaded from the Ocean Color Web operated by NASA (https://oceancolor.gsfc.nasa.gov/). The data included 17 parameters consisting of remote sensing reflectance (*R*_*rs*_*(λ)*) from 10 visible light wavelengths (412, 443, 469, 488, 531, 547, 555, 645, 667, and 678 nm); six environmental parameters derived from *R*_*rs*_ (Chl *a*, diffuse attenuation coefficient for downwelling irradiance at 490 nm (*K*_*d*_(490)), particulate organic/inorganic carbon concentration (POC/PIC), photosynthetically available radiation (PAR), and normalized fluorescence line height (nFLH)); and another environmental parameter, SST, derived from infrared measurements. The data were acquired from January 2003 to December 2021. To reduce the number of missing values, the data from both satellites were used. If the values from both satellites were available for a grid cell, averaged values were used because they were well correlated (Fig. S1).

### Two-dimensional projection of satellite-derived parameters

To capture the range of all possible satellite-derived parameter values, a two-dimensional (2-D) projection of randomly selected grid cells was performed. Twenty thousand grid cells were randomly selected from all the 5ʹ square grids with the probability proportional to the area of each grid. After removing grid cells on land or in coastal regions and those with missing data, 7019 grid cells remained (Fig. S2). A sampling month was randomly selected from 120 months (January 2009 to December 2018) for each grid cell. The satellite-derived parameters for these randomly selected grid cells and months were standardized by subtracting the mean and scaling to unit variance. Finally, the 7019 points with the 17 parameters were projected onto a 2-D map by Uniform Manifold Approximation and Projection (UMAP) using the Python package umap-learn [20].

### Metabarcoding data

Raw sequencing data were downloaded from the EMBL/EBI-ENA EukBank umbrella project in their native format (accession numbers of all BioProjects under the EukBank umbrella project are listed in Data S1). When applicable, reads were merged and trimmed (using vsearch [21] and cutadapt [22]) to cover the 18 S V4 region, as defined by the primers TAReuk454FWD1 and TAReukREV3 [23], resulting in 347,327,830 unique sequences, representing 1,672,099,024 reads. After clustering (swarm [24]), chimera detection (uchime [25]), quality-based filtering, and post-treatments based on occurrence patterns (swarm, lulu [26]; https://github.com/frederic-mahe/mumu), representative sequences were compared with the 18 S rDNA database EukRibo [27], using a global pairwise alignment approach (usearch_global command in vsearch), and taxonomically assigned to their best hit (https://github.com/frederic-mahe/stampa/). The filtered occurrence table of EukBank contained 460,147 operational taxonomic units (OTUs) clustered by swarm, representing 1,403,019,176 reads, collected from 15,562 samples. The sequencing data from the EukBank umbrella project included the amplicon sequence data (837,127,965 reads) targeting 18 S V4 regions from 1011 samples (1191 datasets) collected through the *Tara* Oceans expeditions, which are newly released with this paper (accession numbers in Data S2).

To use the filtered occurrence table of EukBank for the analysis, the raw number of reads was rarefied to 10,000 reads per sample. A total of 1715 samples from the ocean surface (depth < 10 m) with spatiotemporal metadata were retained. These came from several ocean sampling projects, including *Tara* Oceans [1], Malaspina [28], and Australian Microbiome [29]. Occurrences in sequencing replicates from *Tara* Oceans were averaged. Samples from *Tara* Oceans were size fractionated by organism size (e.g. four size fractions: 0.8–5, 5–20, 20–180, and 180–2000 µm), but most samples from other projects were not size fractionated (simply 0.2–3 µm or >0.2 µm). The samples from the four size fractions that mainly targeted piconano-plankton (0.2–3 µm, >0.2 µm, 0.8–5 µm, and >0.8 µm) were relatively similar in taxonomic composition (Fig. S3). These four size fractions were selected for use in this study to maximize the number of samples available for analysis. They were averaged inside each of the 653 bins that matched the 5ʹ square monthly satellite data grids. Although more than one sample from different size fractions, sampling location and time were assigned to a single bin, samples in the same bin were more similar compared with samples from different bins (Fig. S4). Hereafter, we call these bins “samples”.

### Spatial resampling

A total of 653 metabarcoding samples from previous processing were further filtered using the following procedure. Samples with missing satellite data values owing to bad weather or other reasons were removed. Samples from locations where the sea floor was shallower than 200 m were detected using a global relief model [30]. They were removed to keep only open ocean samples [31]. Samples were thinned so that they were separated by a minimum of 200 km, using the R package spThin [32]. This procedure resulted in 177 samples available for analysis (Fig. S5).

### Network inference

OTUs were selected by their occurrence to reduce the number of OTUs to those similar to previous studies that analyzed network structures [33, 34]. Two hundred and eight OTUs with a minimum occurrence larger than 0.2% (20 reads) in at least 10% of samples (18 samples) were retained (Fig. S6). OTU read counts were centered log-ratio-transformed [35]. An ecological network was inferred based on co-occurrence patterns using the Julia package FlashWeave [36] with the settings “heterogeneous = False”, “sensitive = True”, and “alpha = 0.05”, as in previous studies [36, 37]. FlashWeave is a package for detecting direct associations between OTU pairs based on the local-to-global learning framework for causal inference. The nodes in the obtained network were OTUs, and the edges were decided based on direct associations between OTU pairs. Only positive associations (edges) were considered here because most module detection algorithms only allow non-negative networks. The module detection performances of eight algorithms (Fast Greedy, Infomap, Label Propagation, Leading Eigenvector, Leiden, Louvain, Spinglass, and Walktrap) were compared using the R package “igraph” (https://igraph.org/). To measure the structure of the detected module division, we used the modularity index Q as defined by the following equation:

$$Q=\frac{1}{2S}\mathop{\sum}\limits_{u,v}\left(\sigma \left(u,v\right)-\frac{{k}_{u}{k}_{v}}{2S}\right)\delta \left({M}_{u},{M}_{v}\right)$$

where *u*, *v* are nodes (OTUs), $\sigma \left(u,v\right)$ is an edge weight (association strength) between *u* and *v*, *S* is the sum of all edge weights, *k*_*u*_ is a weighted degree of node *u*, *M*_*u*_ is a module to which node *u* belongs, and $\delta \left(x,y\right)$ is 1 if *x* = *y* and 0 otherwise [38].

### Edge satisfaction

We defined an edge satisfaction index to measure the completeness of each module in a sample. If *M* is a module and *i* is a sample, then the edge satisfaction index of *M* and *i* is defined by,

$${{ES}}_{M,i}=\mathop{\sum}\limits_{u,v\in M}\sigma \left(u,v\right)\min \left({p}_{i}\left(u\right),{p}_{i}\left(v\right)\right)/\mathop{\sum}\limits_{u,v\in M}\sigma \left(u,v\right)$$

where *u*, *v* are nodes, $\sigma \left(u,v\right)$ is an edge weight between *u* and *v*, ${p}_{i}\left(u\right)$ is a weight of node *u*, which is the sigmoid transformation of the centered log-ratio-transformed read count of OTU *u* in sample *i*. Briefly, this index measures the ratio of the number of edges between existing nodes in a given sample and the number of all the edges within a given module. The nodes and edges had a weight between 0 and 1 (because only positive associations were considered). The edge satisfaction index was thus also between 0 and 1.

This index was used for the assignment of a community type to each sample. Each community type was defined as a sample in which the corresponding module had the highest edge satisfaction index.

### Machine learning and cross-validation

Several machine learning algorithms were used to train predictive models of the community types from satellite-derived data. Spatial parameters (longitude and latitude) were also tested for their prediction ability. The sine and cosine of the longitude were used as independent parameters because longitude is circular (−180° and 180° are the same). K-nearest Neighbors, Naïve Bayes, Multilayer Perceptron, Random Forest, and Support Vector Machine (SVM) were applied using the Python package “scikit-learn” (https://scikit-learn.org/). In the training process for all the methods, except Random Forest, the satellite-derived and spatial parameters were standardized by subtracting the mean and scaling to unit variance. Both leave-one-out cross-validation and buffered cross-validation [39] were used to measure the model accuracy. In the buffered cross-validation, a test sample was chosen similar to leave-one-out, but samples inside a buffer region surrounding the test sample were excluded from training samples. The buffer was set to a radius of 2000 km from the test sample. In each fold of the training, hyperparameters were chosen through an exhaustive search using the implementation of grid search in scikit-learn. The hyperparameters that were tuned with the grid search are shown in Table S1. The class prediction output of each method was used to measure accuracy, and output probabilities were used to calculate the receiver operating characteristic (ROC) curve.

A predictive model of the community type was constructed by training a machine-learning model with all 177 samples. The machine learning method that recorded the highest performance in cross-validation was used for training. A five-fold grid search was used to choose hyperparameters. The permutation importance of each parameter for the prediction of individual community types was assessed in the obtained predictive model. The permutation importance was calculated as the decrease in the area under the ROC curve (ROC–AUC) when the given parameter was randomly reordered.

### Time series prediction

The constructed model was used to predict the spatiotemporal distribution of each community type based on satellite data. Satellite data mapped to the 5ʹ square monthly grid from January 2003 to December 2021 were used for the prediction. Satellite data were downsized by choosing a grid cell at the center of each 12 × 12 grid to reduce the computational cost. In other words, a grid cell was chosen for every 1° square grid cell. The long-term trend in the areas of predicted community types was tested by the seasonal Mann-Kendall test and its slope was estimated by the seasonal Theil-Sen’s slope estimator using the python package “pymannkendall” [40].

## Results

### Two-dimensional map of points with 17 satellite-derived parameters

**Fig. 1:**
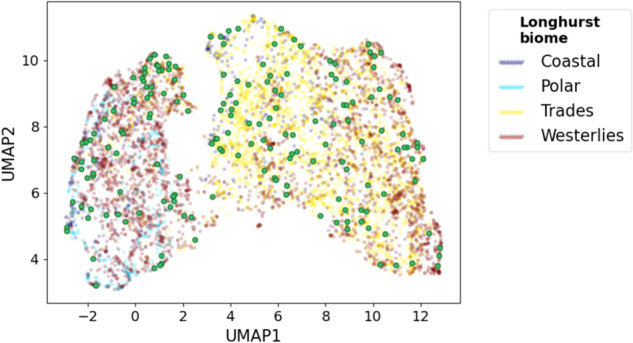
Two-dimensional map of satellite-derived parameter space. Points associated with metabarcoding samples used to train predictive models are projected on the parameter space map (large green points). Small points are randomly selected grid cells, which were used to train a UMAP projection, colored by the Longhurst biomes (see Fig. S7).

We generated a 2-D map of points with 17 satellite-derived parameters using UMAP to observe the parameter ranges (Fig. 1). More than seven thousand points were used to train a UMAP projection. These points were randomly selected from all available locations and times to document the shape of the “continents” in the parameter space map, which represents the possible range of values of the satellite-derived parameters (Figs. S2 and S7). Points associated with the EukBank metabarcoding samples were scattered among all regions in the continents of the parameter space map. We found that the metabarcoding data covered a wide range of parameter space and were suitable for analysis in terms of their relationship with satellite data, although the number of samples was not large.

### Network inference and module detection

**Fig. 2:**
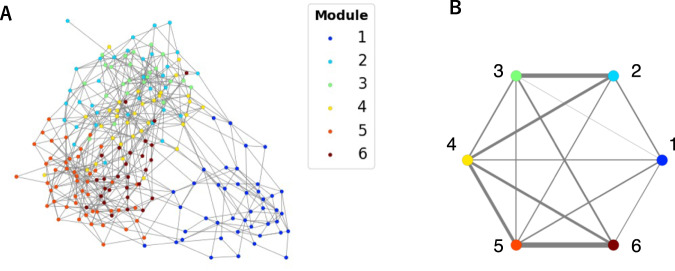
Plankton network inferred using metabarcoding data. **A** Force-directed representation of the network. Nodes (plankton OTUs) are colored by the module they belong to. **B** Connections between modules in the network. The edge width is proportional to the number of inter-module edges.

The ecological network based on OTU co-occurrence patterns was inferred using the FlashWeave algorithm. OTUs were selected by their occurrence (see Materials & Methods). In the network, 560 positive edges (association strength > 0) between 208 OTUs were detected (Fig. 2A). We applied several module detection algorithms to the network. The modules detected by the Leiden and Spinglass algorithms had the highest modularity index (0.55) (Fig. S8). In the following analysis, the modules detected by the Leiden algorithm [41] were used because it captured the macrostructure better than the others (i.e., there were no small modules) (Fig. S8). Among the six detected modules, module 1 was well separated from the other five modules, which formed one super module with a highly aggregated module structure (Fig. 2B). In the super module, modules 2 and 3, 5 and 6 were strongly connected (Fig. 2B).

**Fig. 3:**
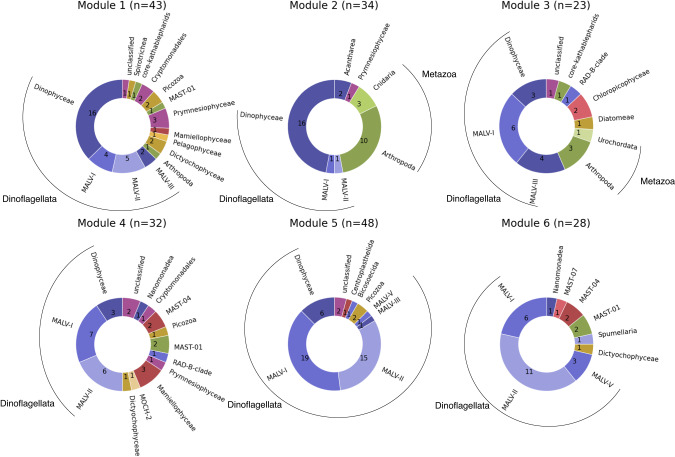
Taxonomic breakdown of modules in the plankton network. The breakdown of taxa annotated to OTUs belonging to each module. The taxonomic level is “taxogroup 2” in the EukRibo.

The taxonomic breakdown of each module is shown in Fig. 3. The well-separated module 1 mainly contained Dinoflagellata (mainly Dinophyceae) as the members, but included Dictyochophyceae (silicoflagellates) and Prymnesiophyceae (haptophytes). The other five modules, which formed the super module, had different characteristics in terms of the taxonomy of the members. Most of the members of modules 5 and 6 were Dinoflagellata (mainly MALV-I and MALV-II), but modules 2 and 3 also contained some Arthropoda (zooplankton) as the members. The read counts of zooplankton OTUs seemed not to reflect the relative abundance of adult animals, but relate to their debris, eggs, or feces, considering the size fractions of samples [42]. The members of module 4 consisted of half Dinoflagellata and half a variety of other taxa. Data S3 contains the taxonomic annotation and assigned module for each OTU.

### Community type of samples

**Fig. 4:**
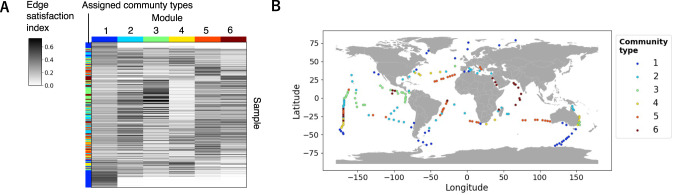
Assigned community types of samples. **A** Heatmap of the edge satisfaction index. The rows are samples ordered by their latitude and the columns are modules. The leftmost column shows the community type of each sample by color. Community types were assigned using the module with the highest edge satisfaction index. **B** Geographic distribution of community types. The community type assigned for each sample is shown in the color of the sampling site on the map.

The newly proposed edge satisfaction index was used to measure the completeness of the network module in each sample (see Materials & Methods). Figure 4A shows the edge satisfaction index of each module in all samples. Notably, module 1 tended to be the only module with a high edge satisfaction index in high-latitude samples. We assigned community types 1–6 to samples in which modules 1–6 had the highest edge satisfaction index, respectively. The geographic distribution of the community types is shown in Fig. 4B. Community type 1 was associated with high-latitude regions, including the Arctic and the Southern Oceans. Community types 3 and 6 were mainly seen in tropical regions of the Pacific and the Indian Oceans, respectively. The other three community types were associated with mid-latitude regions.

In the 2-D map of satellite-derived parameter space, samples formed clusters of community types (Fig. S9). For example, clusters of community types 1 and 5 were located at the bottom of the small and large continents of the parameter space map, respectively. This distribution implies a relationship between the satellite parameters and the community types.

### Prediction performance

**Fig. 5:**
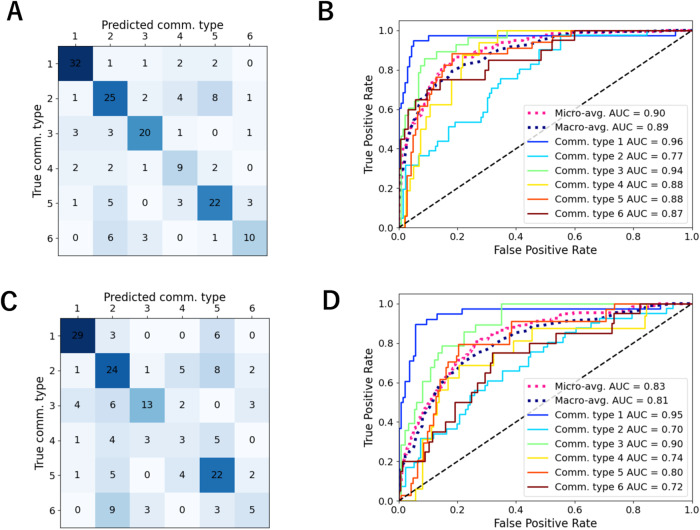
Performance of Support Vector Machine (SVM) on community type prediction using satellite-derived parameters. Performance of SVM using all 17 satellite-derived parameters. **A**, **B** The confusion matrix (**A**) and the ROC curve (**B**) in the leave-one-out cross-validation. **C**, **D** The confusion matrix (**C**) and the ROC curve (**D**) in the buffered cross-validation.

We applied several machine learning algorithms to classify the community types based on satellite-derived parameters. Among the five machine learning methods used, SVM achieved the highest prediction accuracy and micro-average ROC–AUC (Table S2). Using leave-one-out cross-validation, the accuracy and the ROC–AUC of SVM were 0.67 and 0.90, respectively (Fig. 5A, B). Using buffered cross-validation, which excluded the neighbors of a test sample from the training samples, the measures were reduced to 0.54 and 0.83, respectively (Fig. 5C, D).

We compared the prediction performance when different sets of satellite-derived and spatial parameters were used (Table 1, Figs. S10 and S11). For the prediction only using spatial parameters (latitude and sine/cosine of longitude), the ROC–AUC dropped from 0.91 to 0.59 (close to 0.50, i.e., random prediction) when the cross-validation method was changed from ordinary leave-one-out to the buffered one (spatial bias controlled). In contrast, there was a small decrease from 0.90 to 0.83 for the prediction using all 17 satellite-derived parameters. This result demonstrated the advantage of using satellite-derived parameters to classify the community types when spatial biases were appropriately controlled. The prediction performance with only one satellite-derived environmental parameter—SST or Chl *a*—was not as good as the one with all satellite-derived parameters, but it did improve when SST and Chl *a* were combined. Adding the other five satellite-derived environmental parameters (*K*_*d*_(490), POC, PIC, PAR, and nFLH) to SST and Chl *a* further improved the performance but it was still slightly worse than that with all 17 satellite-derived parameters, including *R*_*rs*_.

**Table 1:** Performance of Support Vector Machine (SVM) on community-type prediction when different sets of satellite-derived and spatial parameters were used.

Parameter set	Leave-one-out cross-validation		Buffered cross-validation	
	Accuracy	ROC-AUC^a^	Accuracy	ROC-AUC^a^
All 17 satellite-derived parameters	0.67	0.90	0.54	0.83
Latitude, Longitude^b^	0.68	0.91	0.29	0.59
SST	0.40	0.79	0.28	0.72
Chl *a*	0.43	0.72	0.23	0.62
SST, Chl *a*	0.52	0.86	0.47	0.82
All seven environmental parameters	0.58	0.88	0.50	0.83

^a^Micro-average area under the ROC curve.

^b^Sine and cosine of longitude were used as parameters.

A predictive model of community types was constructed by training SVM with all 177 samples. A five-fold grid search selected the linear kernel and the L2 penalty parameter C = 1.0 for the predictive model. The chosen threshold of the output probability of SVM was 0.28, which gave the highest F1 score in cross-validation (Fig. S12). The importance of each parameter for the prediction of individual community types was assessed in the predictive model (Fig. S13). Only the SST was important in the prediction of community type 1. For other community types, SST and also PAR (community type 2, 3, and 4) and *R*_*rs*_ from several wavelengths (community type 2, 5, and 6) were important in the prediction.

### Time series prediction

**Fig. 6:**
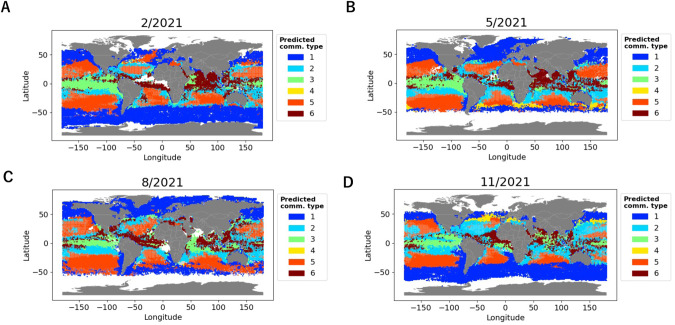
Spatiotemporal distribution of community types predicted from satellite-derived parameters. Community type distribution in February (**A**), May (**B**), August (**C**), and November (**D**), 2021, predicted from satellite-derived parameters. When multiple community types were predicted to the same point, the community type with the highest probability is shown in transparent color. Gray points mean that no community type was predicted.

We applied the obtained model to predict a 19-year time series of community type distribution, from January 2003 to December 2021 (Video S1). The global community type distributions in each season of 2021 are shown in Fig. 6. Community type 1 was mainly in high-latitude regions. Community type 5 predominantly corresponded to the subtropical gyres. Community types 3 and 6 were in tropical regions. Community type 2 filled the gap between community types 5 and 3. Community type 4 showed a pattern related to warm currents. The relationship was to the regions of the Kuroshio and Gulf Stream extensions in the late autumn and early winter in the Northern Hemisphere (November–January) (Fig. 6D, Video S1) and those of the Brazil, Agulhas, and East Australian Currents extensions in the late autumn and early winter in the Southern Hemisphere (May–July) (Fig. 6B, Video S1).

We investigated whether there were long-term trends in the areas of community types for each Longhurst biome (Figs. S14, S15, and S16). Areas with no satellite data showed no trend or a relatively small one. Thus, missing satellite data had only marginal influence in trends in the community-type areas. Most notable trends were seen in the Trades biome. Community type 2 showed a decreasing trend (of 9.77 × 10^5 ^km^2^/year) and community type 6 showed an increasing trend (of 7.89 × 10^5 ^km^2^/year), while SST gradually increased (approximately 0.4 °C) during the period 2003 to 2021 (Figs. S15 and S17). In contrast, community types 1, 3 and 5 showed a relatively small trend in the Trades biome. In particular, the relatively small change in the seasonal rolling mean curve suggested that community types 1 and 5 were stable over the past two decades (Fig. S15). Community type 4 was mainly seen in the Westerlies biome, and its appearance was depressed in the years of 2005 and 2006 (Fig. S16). A decreasing trend in community type 2 and a relatively stable trend in community types 1 and 5 also existed in the Westerlies biome, where SST gradually increased similar to the Trades biome (Figs. S16 and S17). Community type 1 was dominant and stable in the Polar biome, where SST was relatively stable over the observation period (Figs. S14 and S17).

## Discussion

Here, six plankton community types were identified from a global co-occurrence network, and their distribution was successfully predicted from satellite data using a machine-learning approach. The predictive model outputs were plankton community types that were similar to the phytoplankton-dominated community output of the PHYSAT model [15] rather than a quantitative abundance output like the PhytoDOAS model [12, 13]. However, our method has two advantages over these previous models. First, the output of our model was directly connected with the OTUs inferred from the metabarcoding data. We used a swarm for clustering sequences into OTUs, which was designed to maximize taxonomic resolution [24]; thus, the community types integrated high taxonomic resolution information. For example, dinoflagellates were treated as one group in the PhytoDOAS model [12], whereas they were represented by 136 OTUs that were classified into one of the six different modules in this study (Fig. 3 and Data S3). Second, the community-type output from our method can be easily extended. In this study, the network included phytoplankton and heterotrophic protists, but it can be extended to prokaryotes and viruses using their composition data because of their strong association with eukaryotic communities [43, 44]. Prokaryotes and viruses are difficult to observe directly from satellites owing to their small size and lack of optical properties.

Our results indicated that the predictive performance using satellite-derived SST and/or Chl *a* was relatively high (Table 1, Figs. S10 and S11). This was not unexpected because SST and Chl *a* are correlated with microbial community structure in the ocean [1]. We also showed that the predictive performance with all 17 satellite-derived parameters was higher than only with SST and/or Chl *a* (Table 1, Figs. S10 and S11). This result indicated the advantage of using additional environmental parameters (*K*_*d*_(490), POC, PIC, PAR, and nFLH) and *R*_*rs*_ to predict community types, although the improvement of the performance was not large. Hyperspectral *R*_*rs*_ from future global satellite missions such as PACE [45] will likely improve the prediction performance. We used 177 samples (1715 before binning and thinning, see Materials & Methods), which was relatively small for applying a machine learning approach. This may explain why the linear SVM was the best prediction algorithm for our problem. More complex and nonlinear algorithms such as Multilayer Perceptron, Random Forest, and kernel SVM overfitted the training dataset during model training (Fig. S18).

The time series prediction of community types using the constructed model revealed the spatiotemporal distribution of each community type (Fig. 6 and Video S1). Generally, these community-type distributions were similar to previously obtained plankton provinces using the 18 S V4 rDNA dataset of *Tara* Oceans [46, 47]. Those provinces were defined using species compositional dissimilarity between samples, while the community types in our study relied on the species co-occurrence network. The consistent results obtained by different datasets and analytical approaches suggest the stability of plankton community partitioning and corroborate our approach using the newly proposed edge satisfaction to capture community types. Community-type distributions also had some correspondence with the Longhurst biomes [48] (Fig. S7). Community type 1 corresponded with the Polar biome, community type 5 corresponded with the Westerlies biome, and community types 3 and 6 corresponded with the Trades biome. This is consistent with the latitudinal self-organization previously observed and described in plankton community networks [19]. Community type 4 had a seasonal spatiotemporal distribution possibly related to the extensions of the western boundary currents (Fig. 6 and Video S1). A previous study showed that the greatest seasonal changes in environmental variables (phosphate, nitrate, silicate, and dissolved inorganic carbon) occurred in the extension of the Kuroshio among other regions in the Pacific basin [49]. Furthermore, clear seasonal variations in the abundance of cyanobacterial diazotrophs were observed in the same region [50]. Module 4, representing community type 4, connected the two well-connected pairs (modules 2 and 3, 5 and 6) of the super module in the network (Fig. 2B) and had relatively high taxonomic diversity (Fig. 3). In a simulation of emergent phytoplankton in the ocean, areas downstream of the western boundary currents showed high species diversity [51].

Our prediction results identified different long-term trends in areas across community types, which may be related to changes in the SST of the Trades and Westerlies biomes during the observation period. Temperature was the most important environmental factor shaping plankton composition in previous research [1]. Here, our results indicated that the changes (i.e., increase, decrease, or fluctuating trend) in plankton composition at the community level likely reflected the long-term change in SST in the Trades and Westerlies biomes, while community type 5, which corresponded to the subtropical gyres, was relatively stable (Figs. S15, S16, and S17). Consistently, neither the phytoplankton communities nor the temperature showed significant long-term trends in the Polar biome, which was dominated by community type 1, during the observation periods (Figs. S14 and S17). The areas of community type 1 in other biomes, which were related to cold currents from polar regions, were also relatively stable (Fig. S15 and S16). These results imply that our method could detect SST-induced long-term changes in plankton communities that occurred during the past two decades. Notably, the prediction model was only trained on limited sequence data (2009–2017). Therefore, the extrapolations remain to be validated with new sets of sequence data. However, with our cross-validation results, our study underscores the potential to gain insight into complex eukaryotic plankton communities using only satellite data without direct observations.

Although the high taxonomic resolution of metabarcoding data is attractive for research, using a small number of samples imposed several limitations. After binning and thinning, only 177 samples were suitable for our study, although we accessed unprecedentedly large datasets. First, some taxonomic groups were lost in the process of pooling samples to make the analysis dataset. We used only four size fractions, mainly targeting piconano-plankton (0.2–3 µm, >0.2 µm, 0.8–5 µm, and >0.8 µm), to maximize the number of samples available for analysis. Through this procedure, however, taxonomies only observed in larger-size fractions (e.g., diatoms) were lost in the network (Fig. S3A). Second, the network inference algorithm used in this study was not entirely suitable for the dataset. The FlashWeave algorithm had two options: heterogeneous=False for data with a small (hundreds) number of samples from homogenous conditions, and heterogeneous=True for data with a large (thousands and more) number of samples from heterogeneous conditions [36]. We tested both options (Fig. S19), however, it was difficult to judge which option was better because our data were from heterogeneous conditions, but the number of samples was small. We used the results of the heterogeneous=FALSE option for our analysis because this option provided a better performance in predicting known interactions in previous studies [36, 37]. Third, the resolution of the plankton diversity described by six community types was limited. Adding OTUs with relatively low occurrence to the network by changing the cutoff for selecting OTUs (Fig. S6) and tuning the module detection algorithms to capture the microstructure of the network can increase the number of detected modules, which will describe plankton diversity at a higher resolution. However, a machine learning model with a greater number of community types is difficult to train because its prediction performance is dependent on the number of samples for each community type.

In this study, we inferred the ecological network of OTUs using a global metabarcoding dataset and identified six distinct community types of plankton. We applied SVM to construct a predictive model of community types at each site based on satellite data and obtained an accuracy of 67% in cross-validation. The spatiotemporal distribution of community types was shown by applying the model to 19 years of global satellite data at monthly intervals. The study revealed the long-term trends in the distribution of community types, which implied responses to ocean warming. Given the ability of the model to predict the spatiotemporal dynamics of plankton community types from space, our combined network-based and machine-learning approach provides a particularly useful tool to monitor and survey the impact of environmental and climate change on plankton communities at a global scale.

### Supplementary information

Video S1

Supplementary Figures

Supplementary Tables

Data S1

Data S2

Data S3

Supplementary Data and Video Titles

## Data Availability

Figures S1–S19, Tables S1 and S2, Data S1–S3, and Video S1 are provided as supplementary materials. Video S1 shows the 19-year time series of community-type distributions predicted from satellite-derived parameters, related to Fig. 6. Newly sequenced *Tara* Oceans 18 S V4 data have been deposited to EMBL/EBI-ENA: PRJEB6610 (*Tara* Oceans), PRJEB9737 (TARA Oceans Polar Circle). Data and codes used in the analysis are available at the GenomeNet FTP: https://www.genome.jp/ftp/db/community/tara/Satellite/. Essential codes are also available at the GitHub repository: https://github.com/hirotokaneko/plankton-from-satellite.
