# Supplementary material for: Predicting global distributions of eukaryotic plankton communities from satellite data
Source: ISME Commun. 2023 Sep 22;3:101. doi: 10.1038/s43705-023-00308-7 (PMC10517053; doi:10.1038/s43705-023-00308-7)
Supplement: Supplementary file 2 — Supplementary Figures [file 43705_2023_308_MOESM2_ESM.pdf]

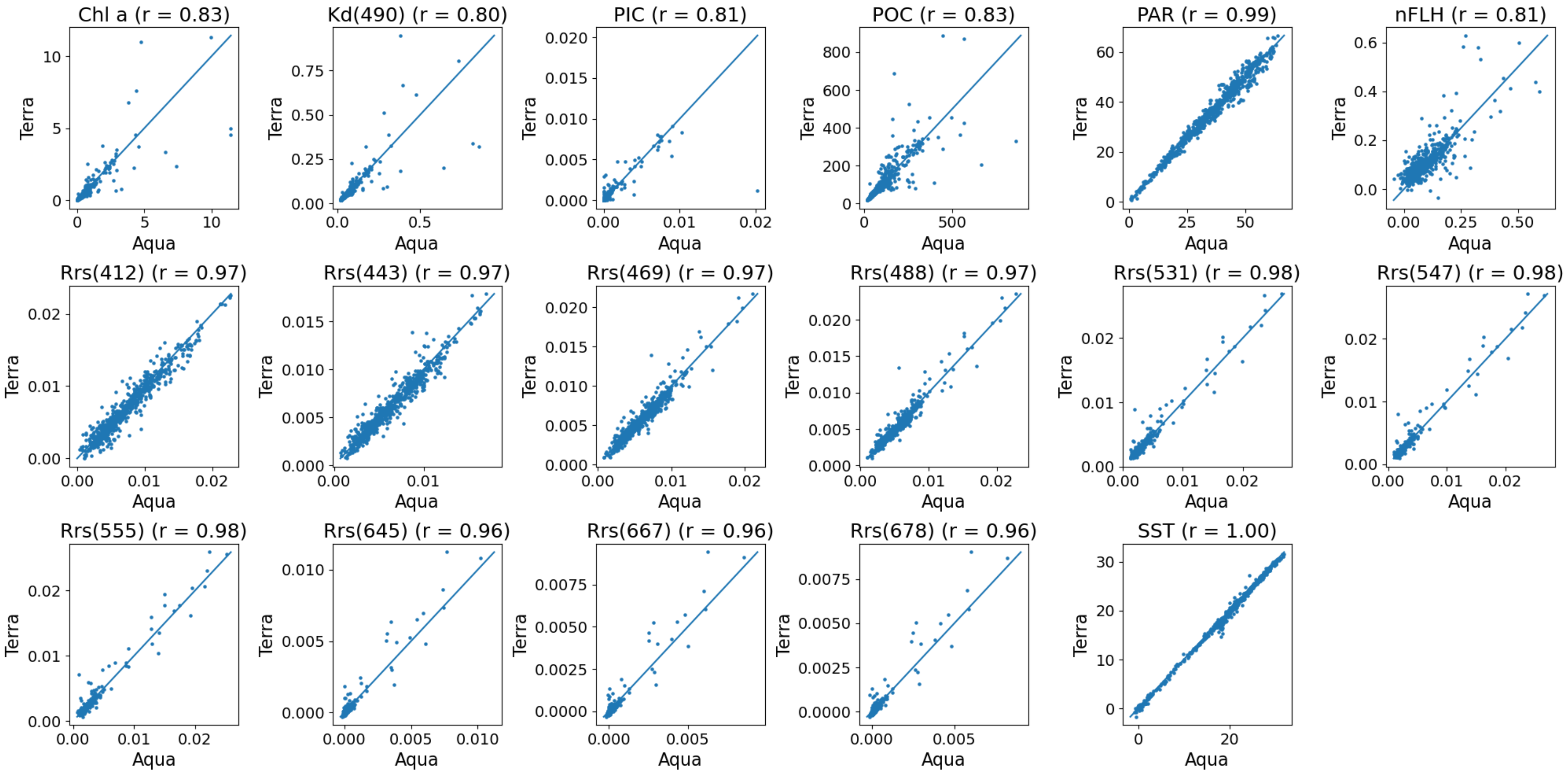

Figure S1. Comparison of satellite-derived parameters acquired by the Aqua and the Terra satellite.
Pearson's correlation coefficient was shown for each parameter pairs.

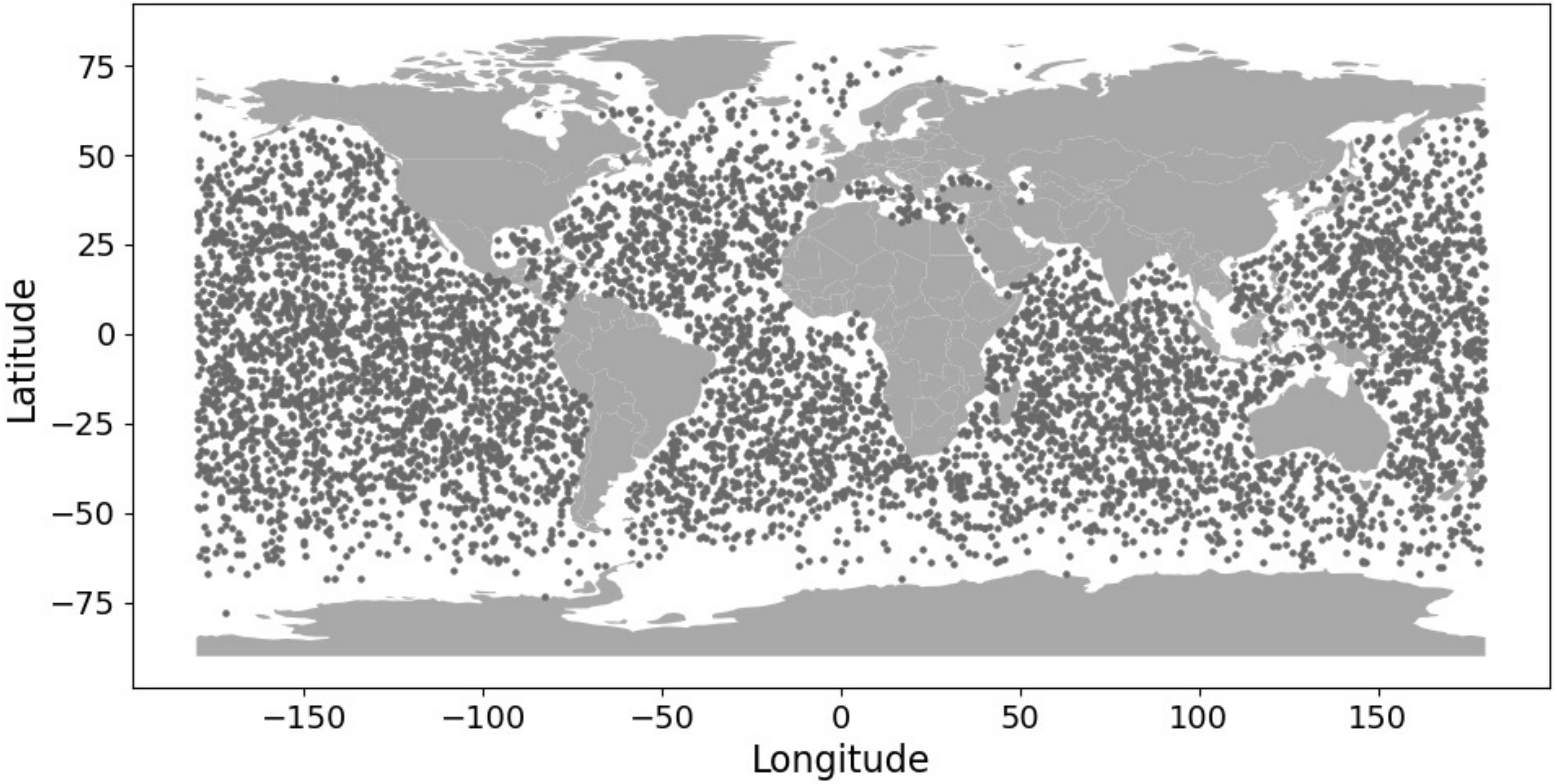

Figure S2. Randomly selected grid cells used to train a UMAP projection.
Dark gray points are location of randomly selected grid cells. Sampling month was also randomly selected for each grid cell.

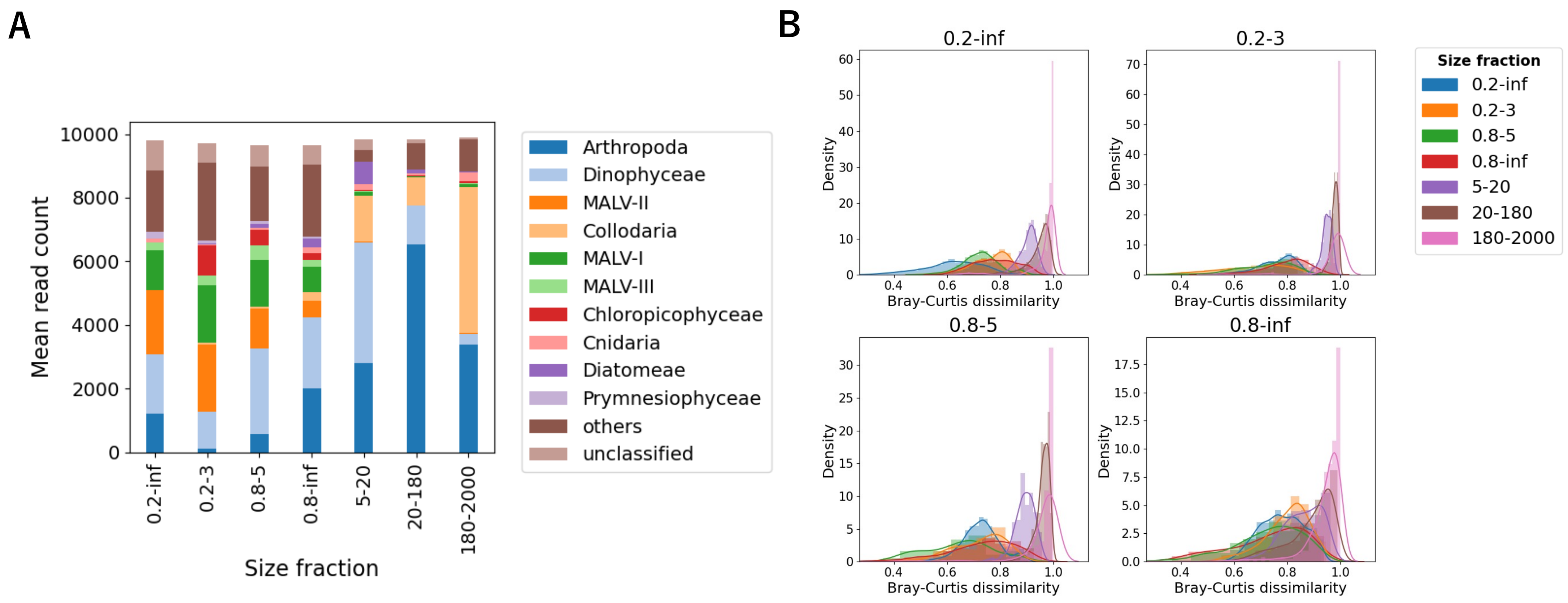

Figure S3. Comparison of size fractions in the South Pacific Subtropical Gyre. Taxonomic difference among size fractions was examined for samples from the South Pacific Subtropical Gyre, which included samples from all major size fractions. **A** Mean taxonomic composition of each size fraction. Taxonomic level is “taxogroup 2” in the EukRibo. **B** Taxonomic dissimilarity of intra- and inter-size-fraction sample pairs. Bray-Curtis dissimilarity was calculated based on OTU read counts.

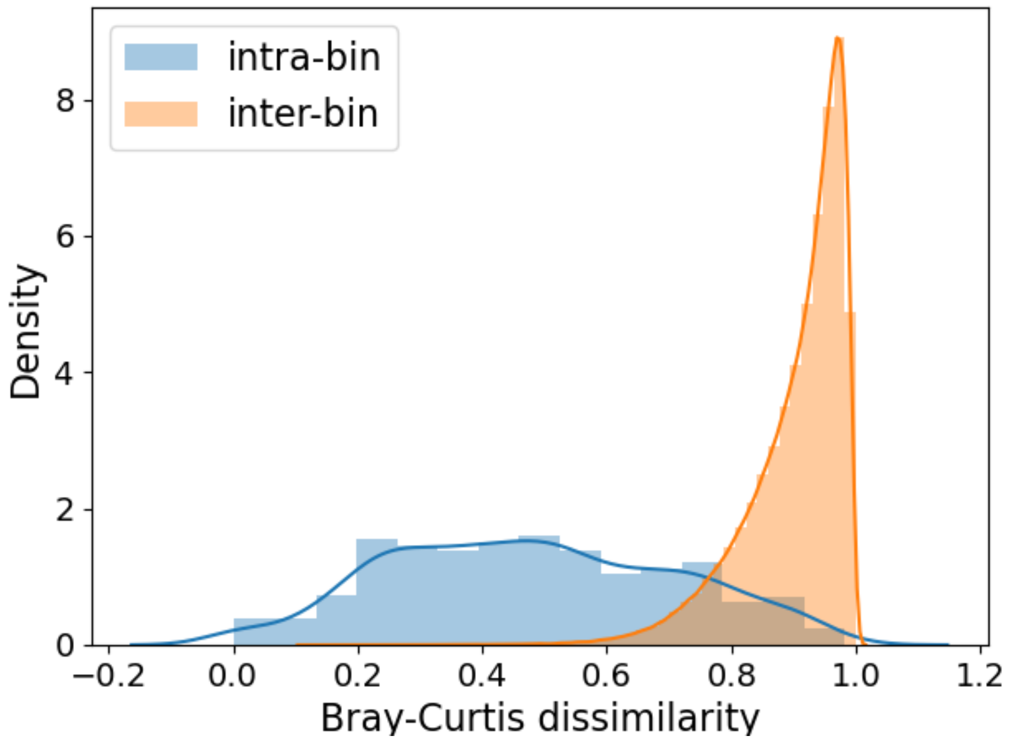

Figure S4. Comparison of dissimilarity of intra- and inter-bin sample pairs.

Bray-Curtis dissimilarity was calculated based on OTU read counts. Intra-bin sample dissimilarity was small enough compared to inter-bin sample dissimilarity in the most cases.

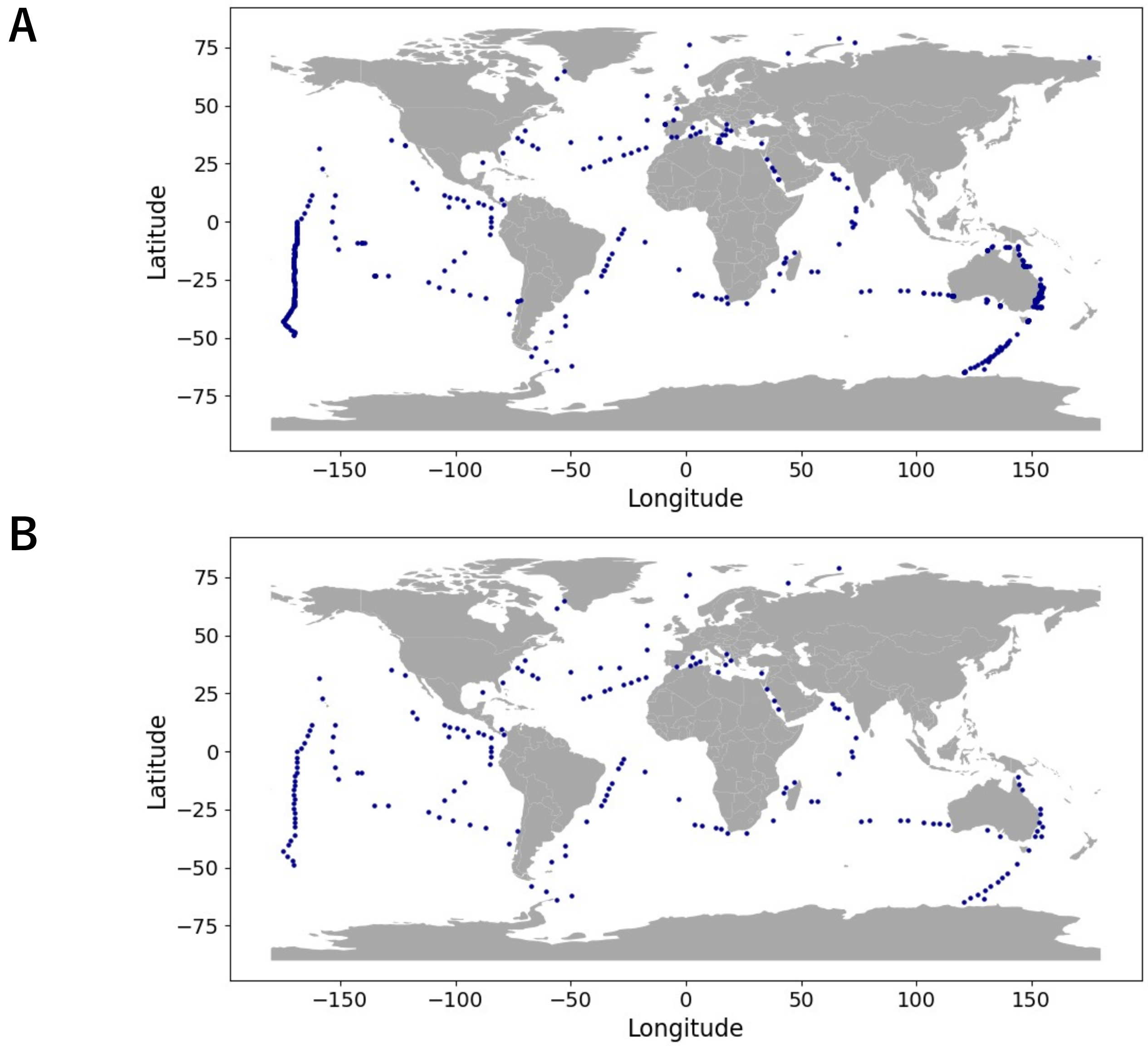

Figure S5. Spatial resampling of metabarcoding data.
A Geographic location of 653 metabarcoding samples (bins) before spatial resampling. **B** 177 samples retained and used for the analysis.

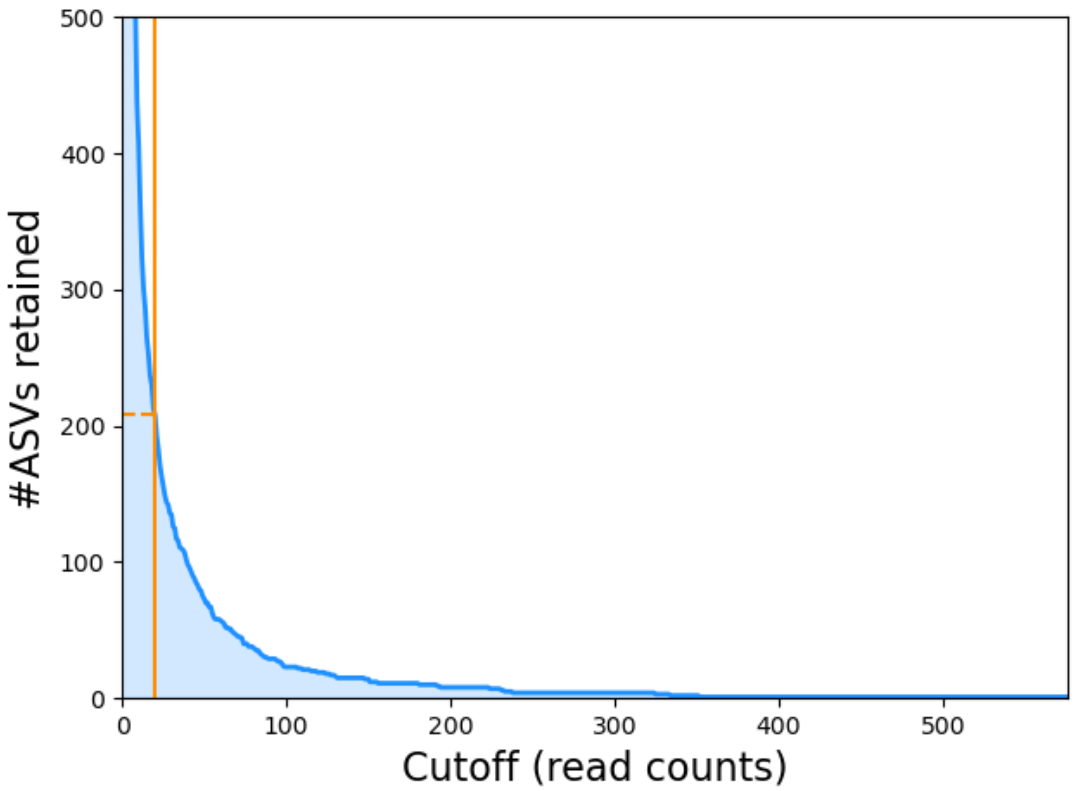

Figure S6. Number of OTUs retained with changing occurrence cutoff.
Blue curve shows the number of OTUs retained with given cutoff used for selection. Orange line is the chosen cutoff (20 reads).

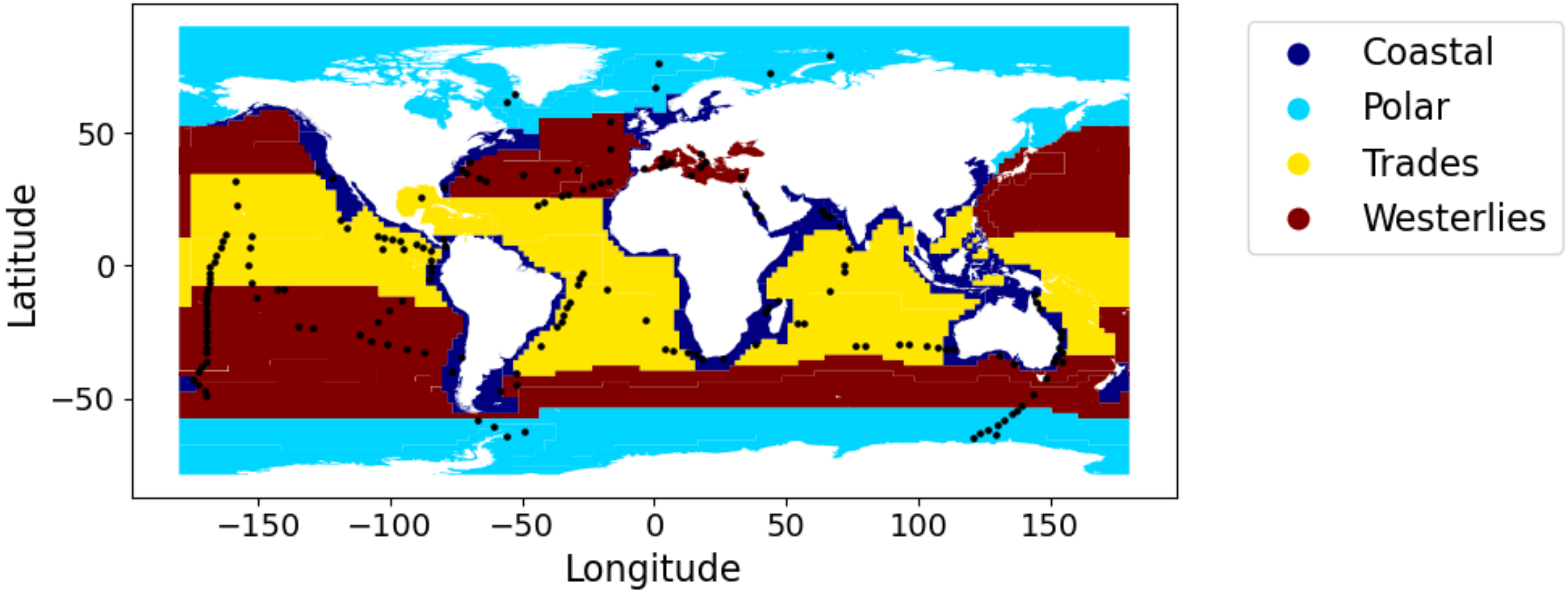

Figure S7. Map of the Longhurst biomes.
Black points are metabarcoding samples. The shape file of the Longhurst biomes was downloaded from Marine Regions (https://www.marineregions.org).

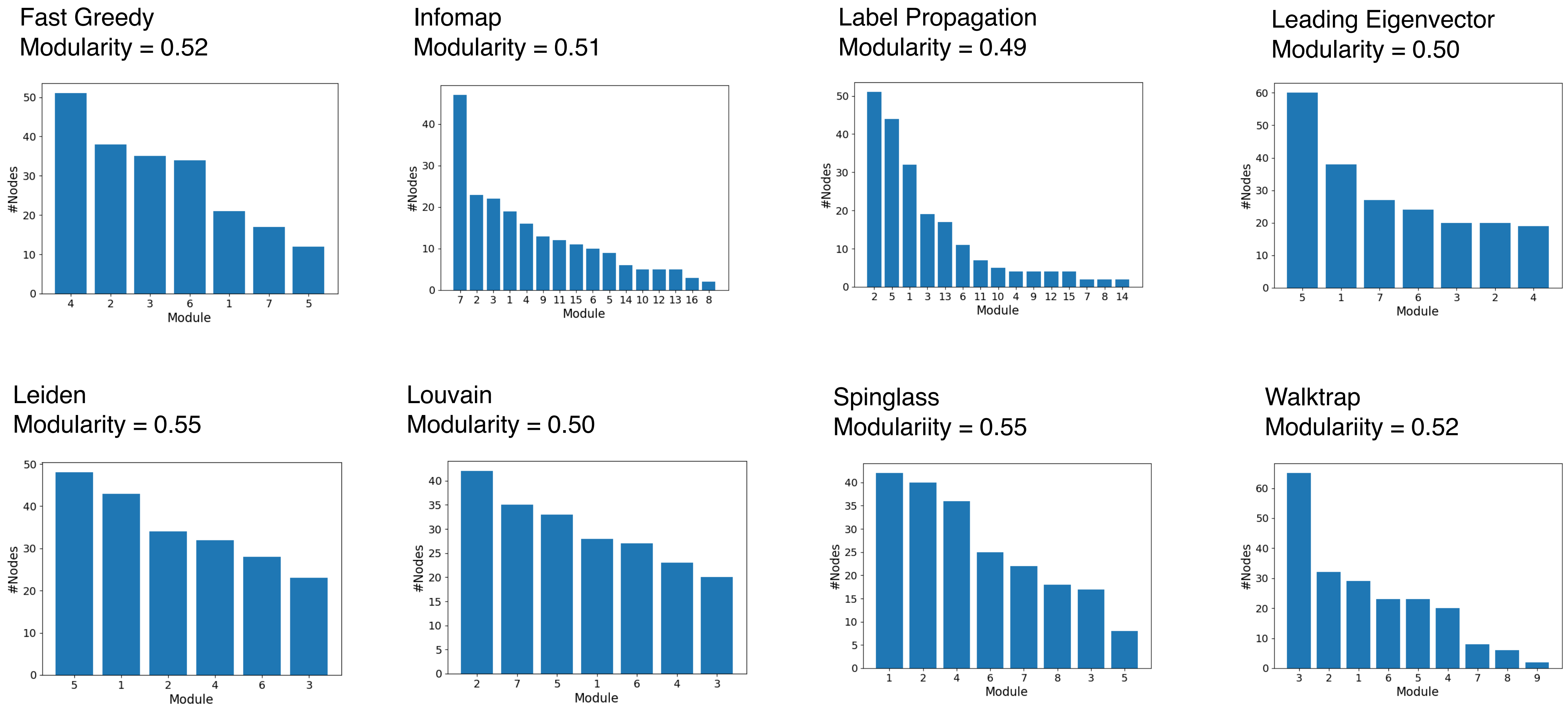

Figure S8. For each algorithm, modularity index and size of the modules.
Modularity index of the module division by each algorithm and size of detected modules by it.

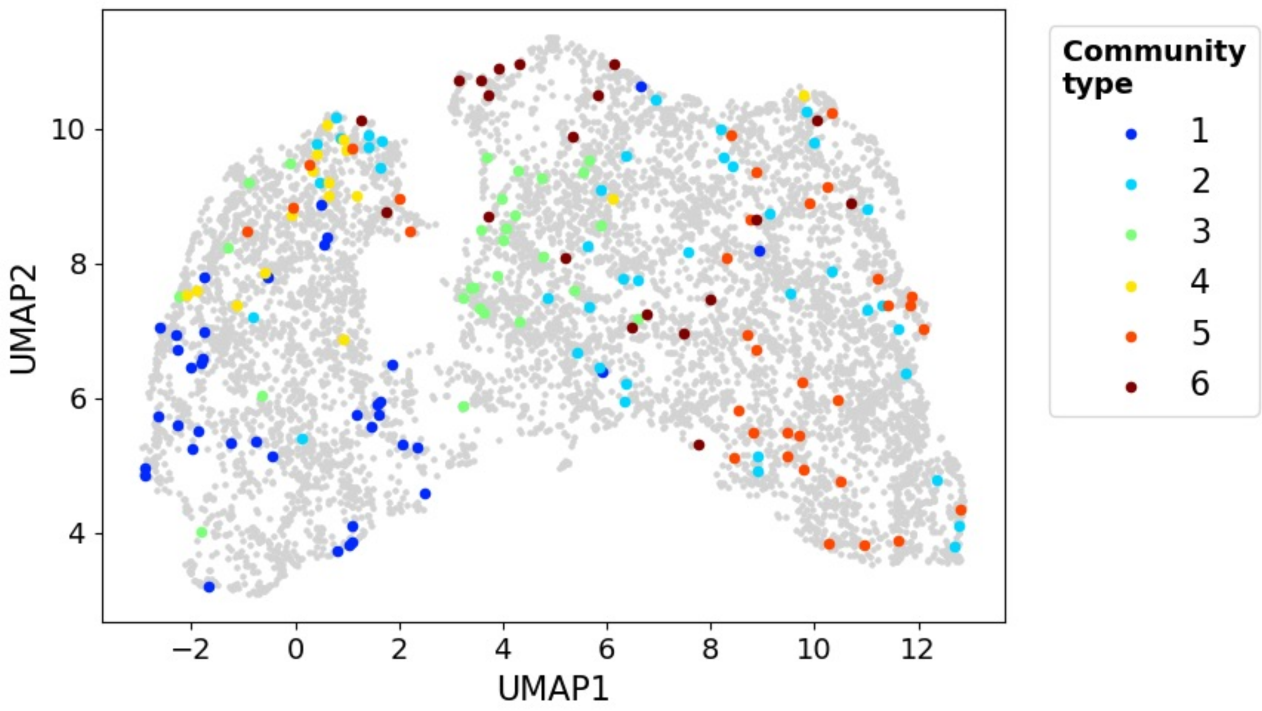

Figure S9. Community type distribution in the satellite-derived parameter space.

Metabarcoding samples projected on the 2-D map of the satellite-derived parameter space colored by community types. Small gray points are randomly selected grid cells used to train a UMAP projection.

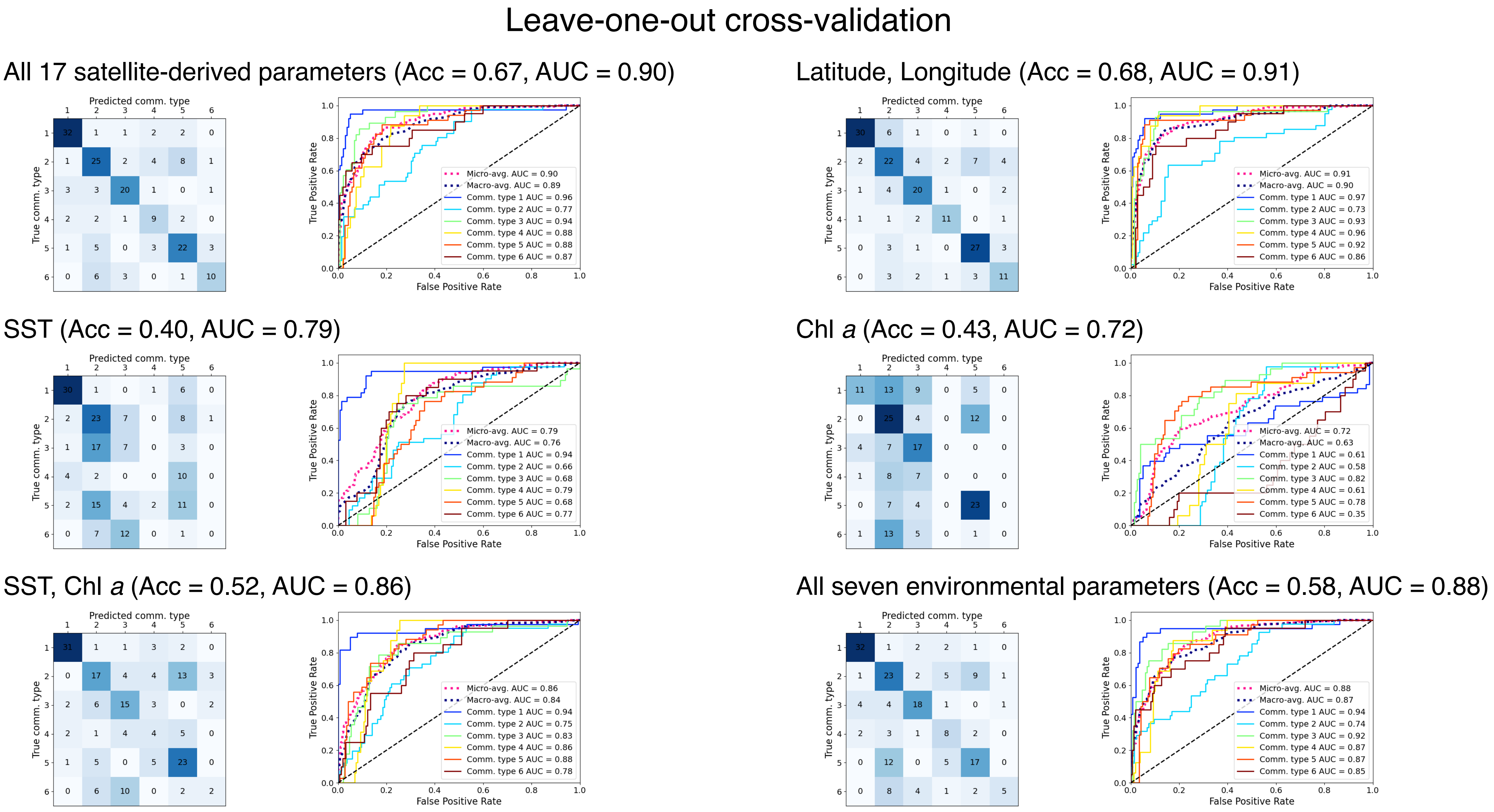

Figure S10. The leave-one-out cross-validation confusion matrix and ROC curve of SVM on community type prediction when different sets of parameters were used, related to Table 1.

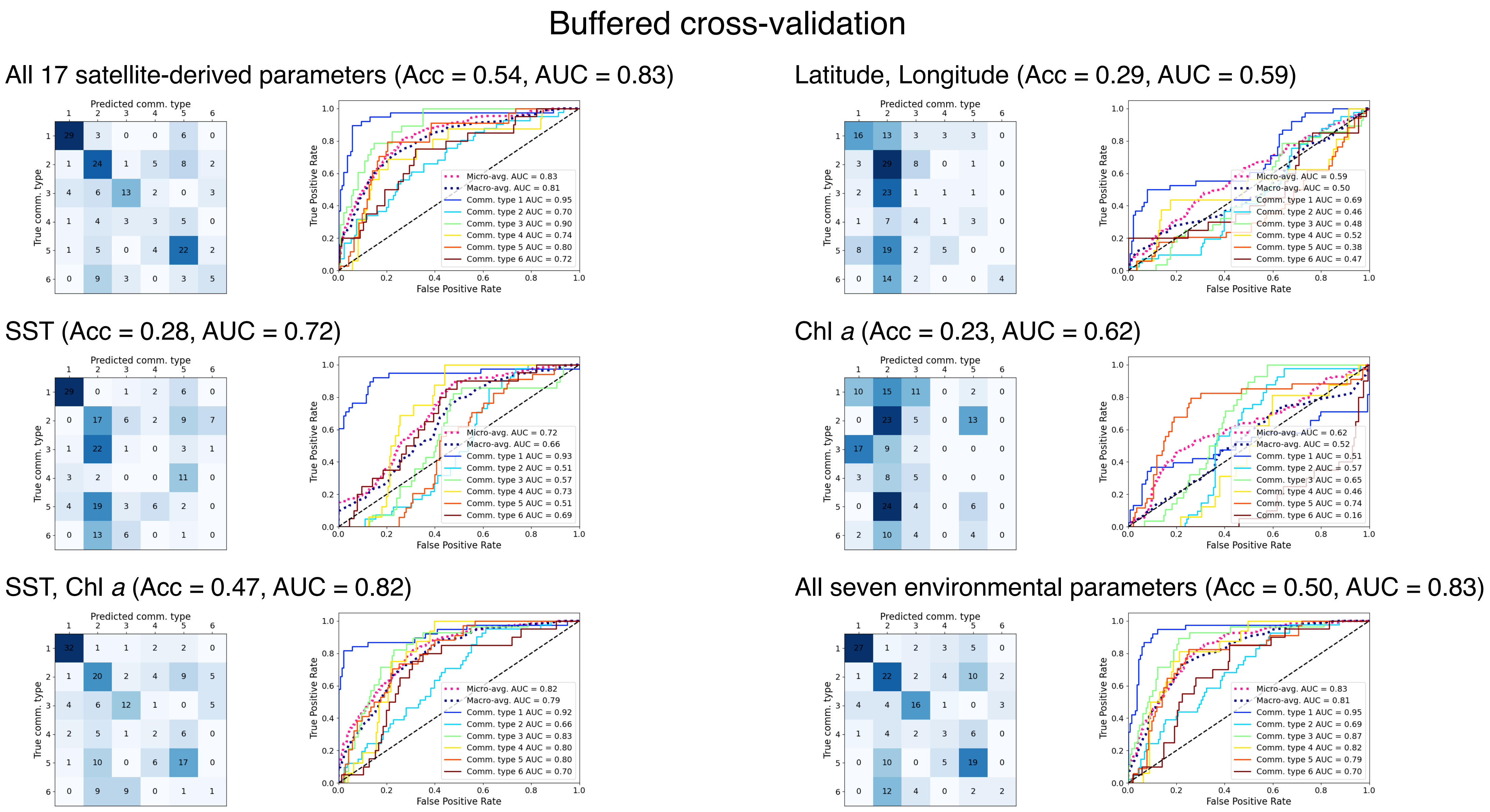

Figure S11. The buffered cross-validation confusion matrix and ROC curve of SVM on community type prediction when different sets of parameters were used, related to Table 1.

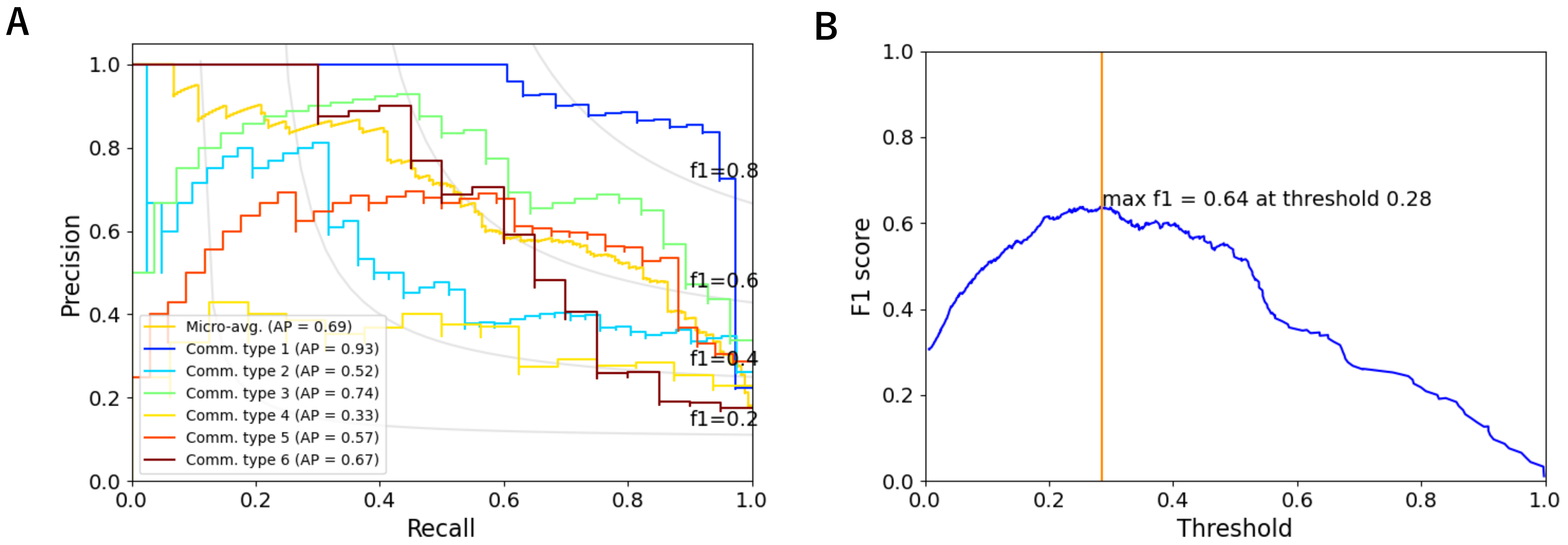

Figure S12. Precision, recall, and F1 score of SVM on community type prediction using all 17 satellite-devired parameters.

A The precision-recall curve in the condition of leave-one-out cross-validation same as Figure 4B. **B** F1 score versus threshold of probabilistic output of SVM. Orange line shows the threshold making highest F1 score.

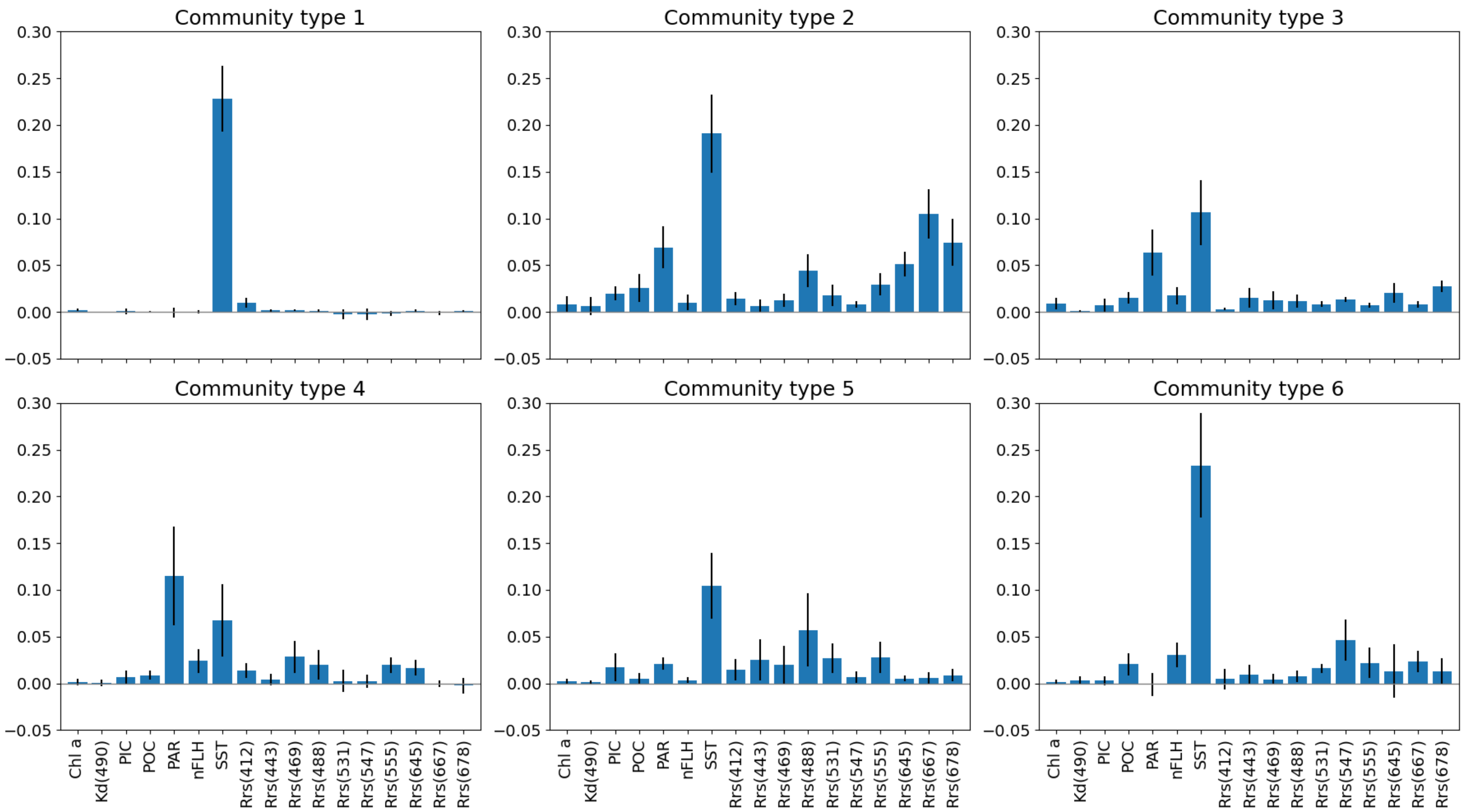

Figure S13. Permutation importance of each parameter for individual community type prediction.

Blue bars show mean values of parameter importance over 5 times repeats (error bars: standard deviation). ROC-AUC was used for scoring.

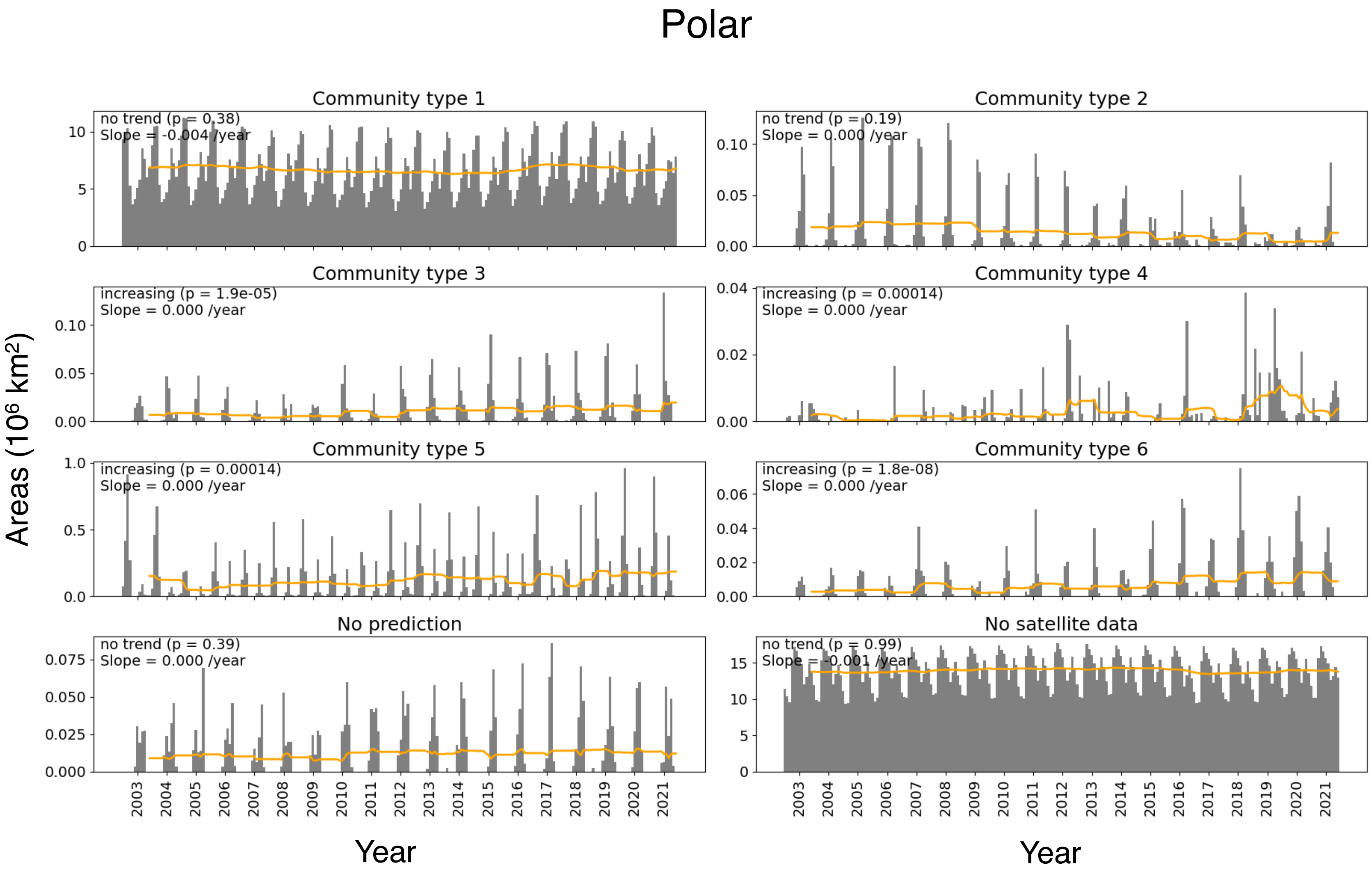

Figure S14. Areas of community types in Polar biome predicted based on satellite data from 2003 to 2021.

Gray bars show the areas in each month and orange lines show the seasonal rolling mean curve of them. Trends were tested by the seasonal Mann-Kendall test and slopes were estimated by the seasonal Theil-Sen's slope estimator.

Trades

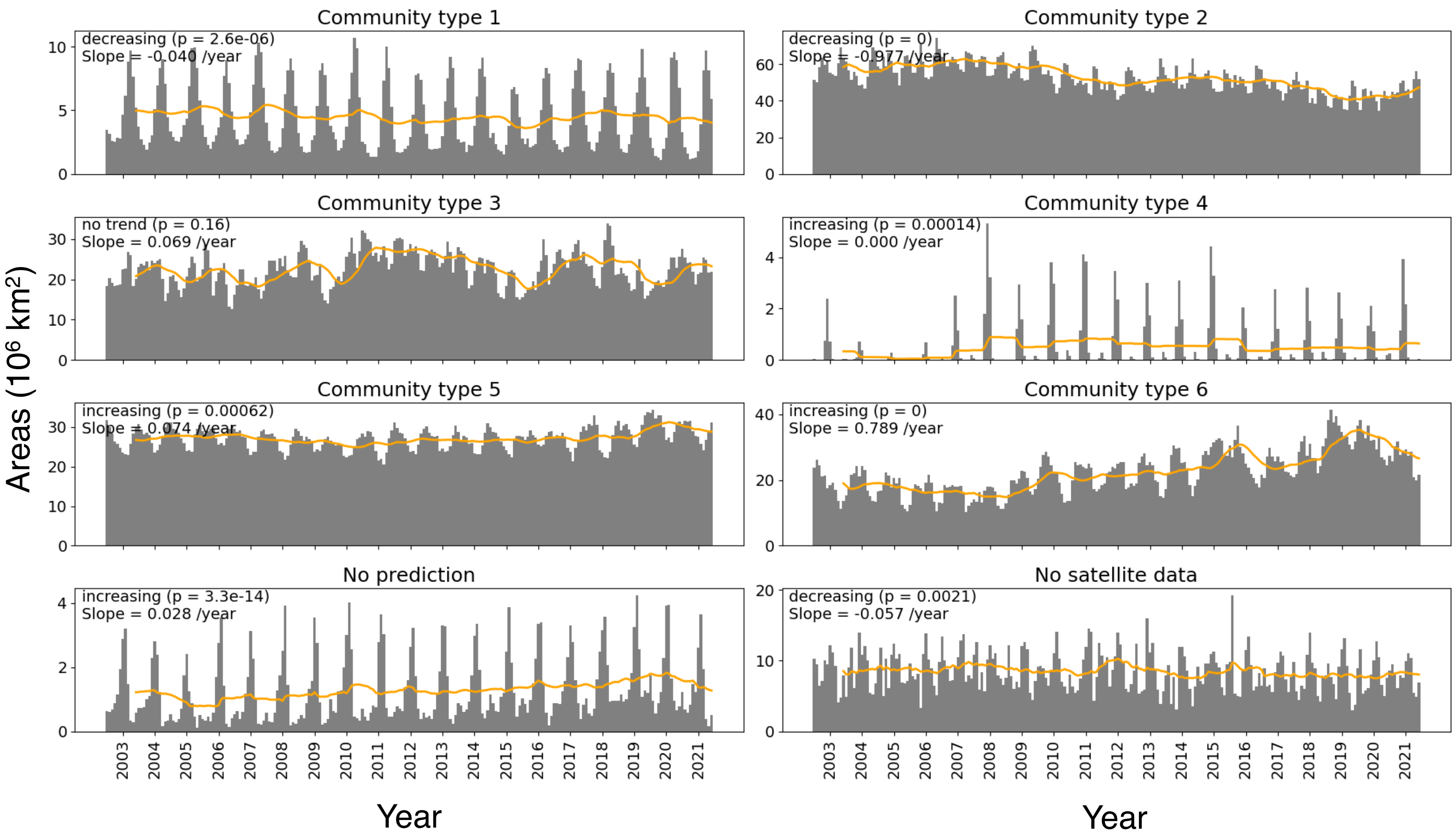

Figure S15. Areas of community types in Trades biome predicted based on satellite data from 2003 to 2021.

Gray bars show the areas in each month and orange lines show the seasonal rolling mean curve of them. Trends were tested by the seasonal Mann-Kendall test and slopes were estimated by the seasonal Theil-Sen's slope estimator.

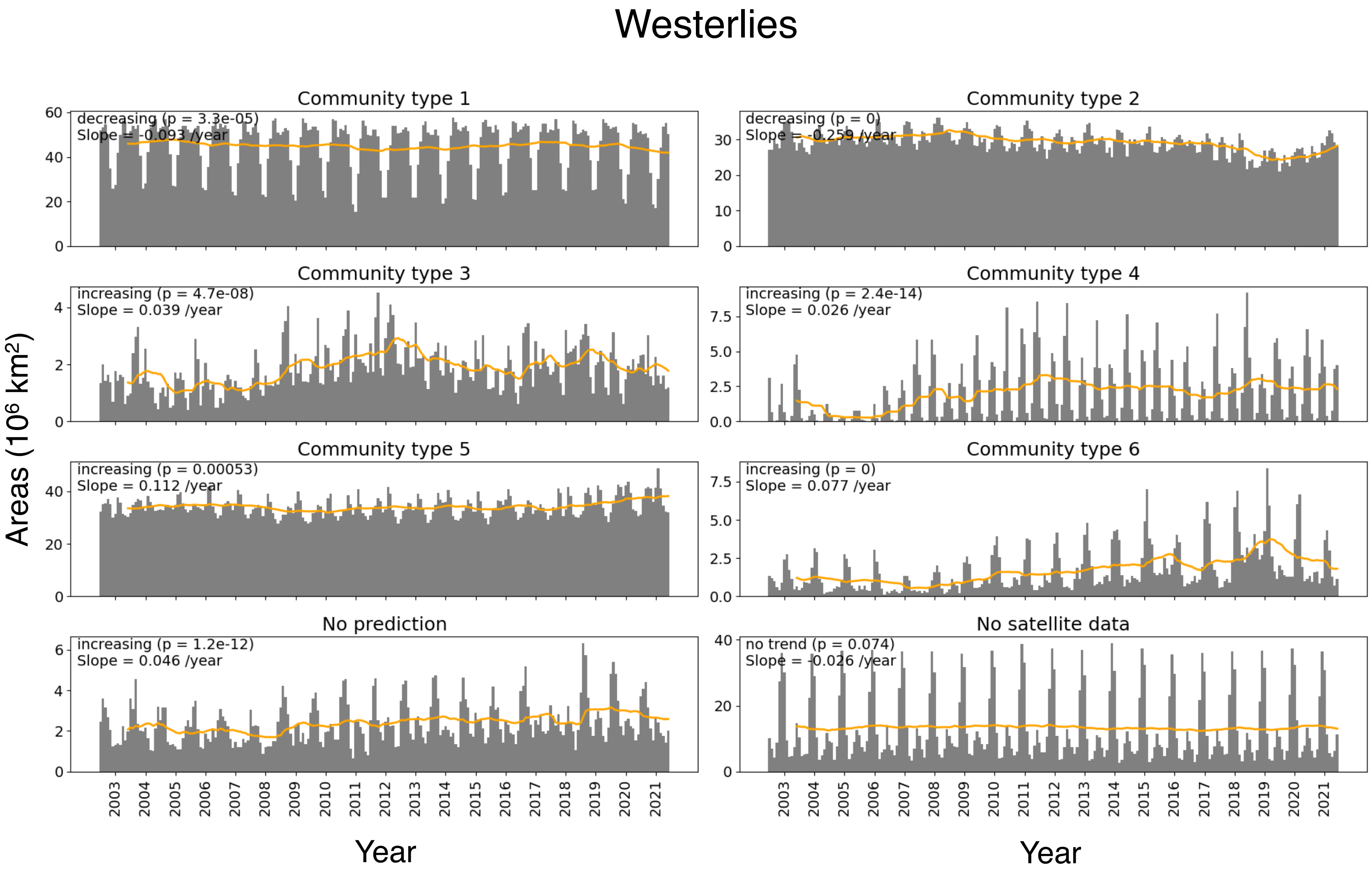

Figure S16. Areas of community types in Westerlies biome predicted based on satellite data from 2003 to 2021.

Gray bars show the areas in each month and orange lines show the seasonal rolling mean curve of them. Trends were tested by the seasonal Mann-Kendall test and slopes were estimated by the seasonal Theil-Sen's slope estimator.

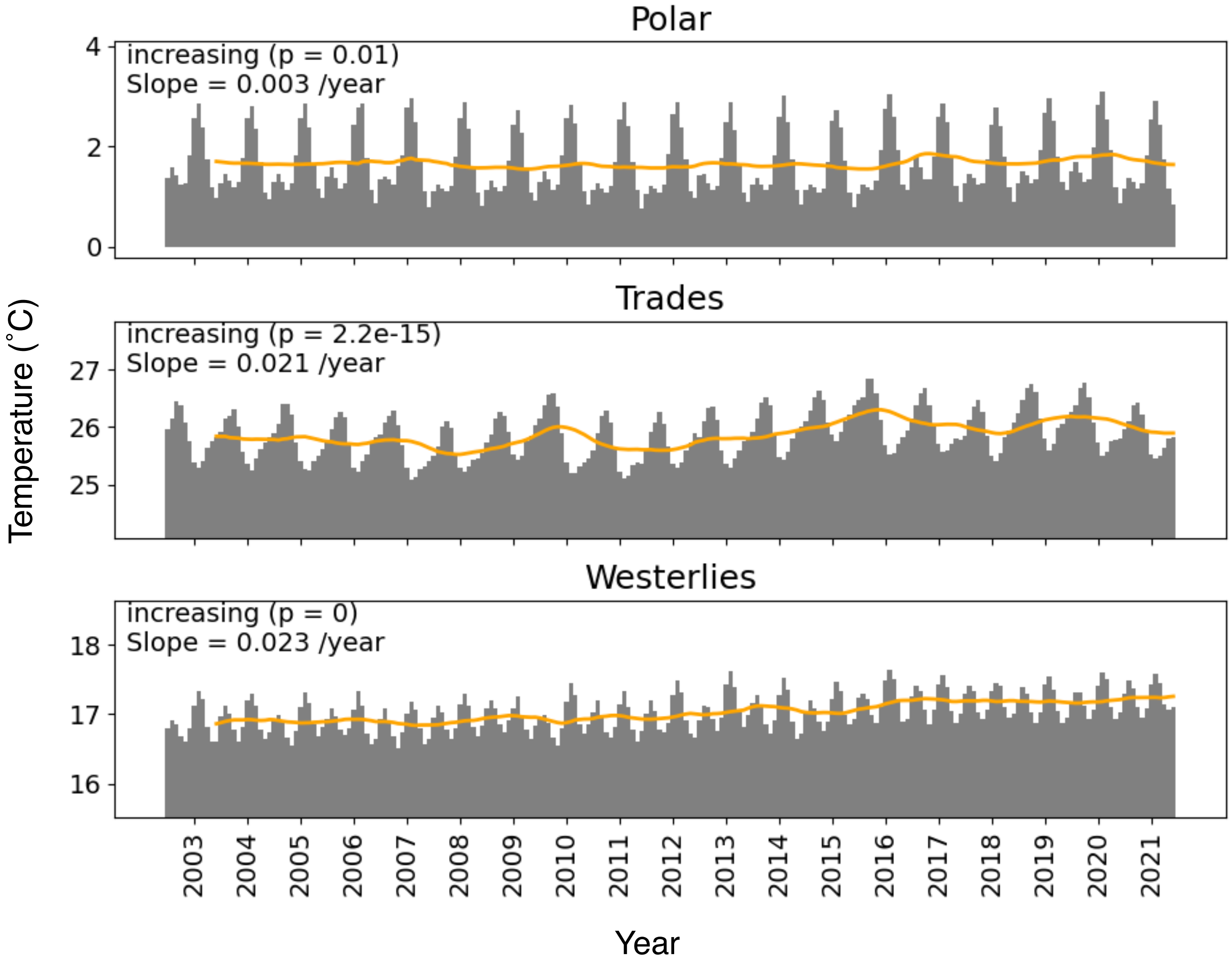

Figure S17. Average sea surface temperature (SST) in each biome observed by satellite from 2003 to 2021.

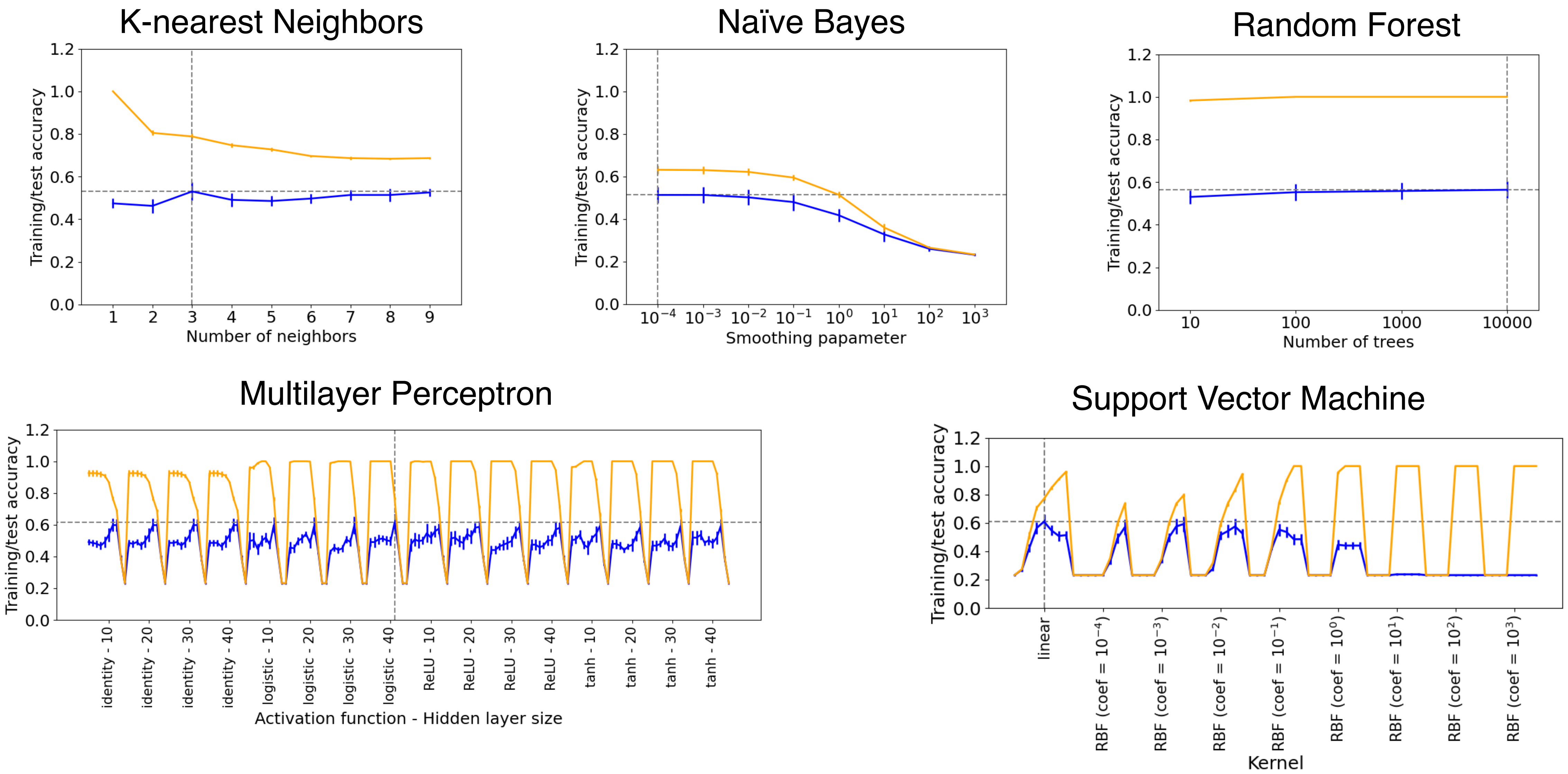

Figure S18. Grid search results in the training of predictive models with all samples. Orange and blue lines show training and test accuracy, respectively. Gray dashed lines show the parameter with the best test accuracy. Ten L2 penalty parameters (10^{-6}, 10^{-5}, …, 10^{3}; from left to right) were tested for each setting of Multilayer Perceptron and eight (10^{-4}, 10^{-3}, …, 10^{3}) were tested for Support Vector Machine.

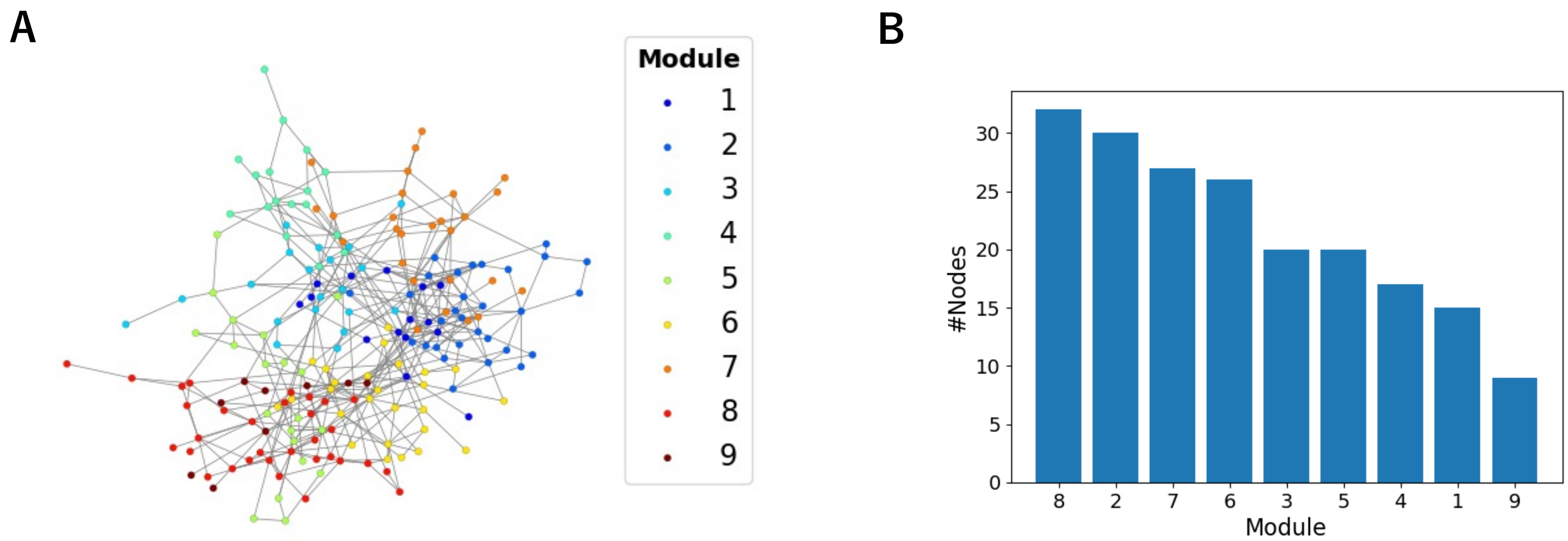

Figure S19. Plankton network inferred with a “heterogeneous=True” option in FlashWeave.
A A force-directed representation of the network. Nodes (plankton OTUs) are colored by belonging module. 415 positive edges (correlation coefficients > 0) between 196 OTUs were detected. Twelve nodes were not included in the network because they had no edges. **B** Size of detected modules by Leiden algorithm. Modularity index was 0.57.
