# Supplementary material for: Predicting global distributions of eukaryotic plankton communities from satellite data
Source: ISME Commun. 2023 Sep 22;3:101. doi: 10.1038/s43705-023-00308-7 (PMC10517053; doi:10.1038/s43705-023-00308-7)
Supplement: Supplementary file 3 — Supplementary Tables [file 43705_2023_308_MOESM3_ESM.pdf]

Table S1. Machine learning methods and their hyperparameters.

Method	Scikit-learn implementation	Hyperparameters
K-nearest Neighbors	sklearn.neighbors.KNeighborsClassifier	Number of neighbors (1, 2, …, 9)
Naïve Bayes (Gaussian)	sklearn.naive_bayes.GaussianNB	Smoothing papameter (10^{-4}, 10^{-3}, …, 10^{3})
Multilayer Perceptron (one hidden layer)	sklearn.neural_network.MLPClassifier	Activation function (identity, logistic, tanh, ReLU), Hidden layer size (10, 20, 30, 40), L2 penalty parameter (10^{-6}, 10^{-5}, …, 10^{3})
Random Forest	sklearn.ensemble.RandomForestClassifier	Number of trees (10, 100, 1000, 10000)
Support Vector Machine	sklearn.svm.SVC	Kernel (linear, RBF), Coefficient of RBF kernel (10^{-4}, 10^{-3}, …, 10^{3}), L2 penalty parameter (10^{-4}, 10^{-3}, …, 10^{3})

Table S2. Performance of machine learning methods on community type prediction using all 17 satellite-derived parameters.

Method	Leave-one-out cross-validation		Buffered cross-validation	
	Accuracy	ROC-AUC[a]	Accuracy	ROC-AUC[a]
K-nearest Neighbors	0.56	0.86	0.47	0.76
Naïve Bayes	0.55	0.85	0.50	0.81
Multilayer Perceptron	0.60	0.88	0.54	0.82
Random Forest	0.63	0.89	0.50	0.81
Support Vector Machine	0.67	0.90	0.54	0.83

a: Micro-average area under the ROC curve.
