# Supplementary material for: Predicting global distributions of eukaryotic plankton communities from satellite data
Source: ISME Commun. 2023 Sep 22;3:101. doi: 10.1038/s43705-023-00308-7 (PMC10517053; doi:10.1038/s43705-023-00308-7)
Supplement: Supplementary file 7 — Supplementary Data and Video Titles [file 43705_2023_308_MOESM7_ESM.docx]

**Supplementary Data and Video Titles**

**Data S1. Accession numbers of all BioProjects under the EukBank umbrella project.**

**Data S2. Accession numbers of the amplicon sequence data targeting 18S V4 regions from samples collected through *Tara* Oceans expeditions.**

**Data S3. Taxonomic annotation and assigned module for each OTU.**

**Video S1. Nineteen-year time series of community type distributions predicted from satellite-derived parameters, related to Fig. 6.**
